# Transcription factors, coregulators, and epigenetic marks are linearly correlated and highly redundant

**DOI:** 10.1371/journal.pone.0186324

**Published:** 2017-12-07

**Authors:** Tobias Ahsendorf, Franz-Josef Müller, Ved Topkar, Jeremy Gunawardena, Roland Eils

**Affiliations:** 1 Division of Theoretical Bioinformatics, German Cancer Research Center, Heidelberg, Baden-Württemberg, Germany; 2 Institute of Pharmacy and Molecular Biotechnology, Bioquant, University of Heidelberg, Heidelberg, Baden-Württemberg, Germany; 3 Department of Systems Biology, Harvard Medical School, Boston, Massachusetts, United States of America; 4 Zentrum für Integrative Psychiatrie, Kiel, Germany; 5 Harvard College, Boston, Massachusetts, United States of America; Pohang University of Science and Technology, REPUBLIC OF KOREA

## Abstract

The DNA microstates that regulate transcription include sequence-specific transcription factors (TFs), coregulatory complexes, nucleosomes, histone modifications, DNA methylation, and parts of the three-dimensional architecture of genomes, which could create an enormous combinatorial complexity across the genome. However, many proteins and epigenetic marks are known to colocalize, suggesting that the information content encoded in these marks can be compressed. It has so far proved difficult to understand this compression in a systematic and quantitative manner. Here, we show that simple linear models can reliably predict the data generated by the ENCODE and Roadmap Epigenomics consortia. Further, we demonstrate that a small number of marks can predict all other marks with high average correlation across the genome, systematically revealing the substantial information compression that is present in different cell lines. We find that the linear models for activating marks are typically cell line-independent, while those for silencing marks are predominantly cell line-specific. Of particular note, a nuclear receptor corepressor, transducin beta-like 1 X-linked receptor 1 (*TBLR1*), was highly predictive of other marks in two hematopoietic cell lines. The methodology presented here shows how the potentially vast complexity of TFs, coregulators, and epigenetic marks at eukaryotic genes is highly redundant and that the information present can be compressed onto a much smaller subset of marks. These findings could be used to efficiently characterize cell lines and tissues based on a small number of diagnostic marks and suggest how the DNA microstates, which regulate the expression of individual genes, can be specified.

## Introduction

The decision to transcribe genes relies on DNA sequence information, which is interpreted by transcription factors (TFs) or other sequence-specific DNA-binding proteins. In eukaryotes, TFs interact with a variety of mechanisms that reorganize chromatin structure, remodel nucleosomes, recruit coregulators, methylate DNA, and post-transcriptionally modify histones and regulatory proteins to collectively regulate transcription. These genetic and epigenetic mechanisms “mark” regulatory loci to yield DNA microstates, a term derived from thermodynamics [1], which essentially considers any particular binding configuration of TFs and histone modification and DNA methylation patterns etc. that may arise at any time point at the regulatory loci of a gene of interest (promoter, enhancers, etc.). In previous work, we showed how the functional relationship between the concentrations of TFs and the level of expression of a gene, also known as the gene regulation function, can be calculated by determining the relevant microstates and the rates of transition between them [1].

In this study, we focus on the structure of DNA microstates, whose combinatorial complexity is potentially enormous. For example, the histone proteins H2A, H2B, H3, and H4 can be modified at as many as 160 different sites. If these modifications were merely binary, as in the case of phosphorylation, this would result in 2^160^ ≈ 10^48^ potential modification patterns on a single nucleosome and vastly more when all the other marks are considered. Only a small fraction of these microstates are observed in practice, implying associations between different marks and high levels of redundancy between them. We pursue several questions: How strong are these associations? Can most marks be predicted by knowing only a few? Are the rules of association specific to particular cell lines or do they hold generally across many different cell lines?

The large-scale data emerging from consortia like ENCODE [2] and the NIH Roadmap Epigenomics project [3] have provided an opportunity to address these questions. These data measure a variety of epigenetic and regulatory protein marks over the whole genome at steady state for many cell lines/types. Rules of association have been sought using Bayesian networks [4–6], hidden Markov models [7, 8], and other methods [9–11], including some that also incorporate gene expression data [12–14]. While these approaches have shown utility for the efficient prediction and imputation of other marks, none of them uses completely linear models for jointly studying epigenetic marks, TFs, coregulators, and chromatin remodelers. Hence, the question remains, if the correlation structure for all these marks underlies, in fact, linear characteristics, which in turn would lead to models that are easy to interpret in a biological context.

Here, we combine data for epigenetic marks with data for TFs, coregulators, and chromatin remodelers and avoid discretization to enhance sensitivity. We find that simple linear models capture strong associations among these marks within a cell line, with a small subset of marks being able to predict most other marks with high average correlation across the genome, which means here at protein coding and lincRNA genes. We further show that these linear models are largely cell line-independent for activating marks and largely cell line-specific for silencing marks. Our results suggest how cell lines can be characterized by epigenetic and regulatory protein marks and improve our understanding of gene regulation.

## Results

**Fig 1:**
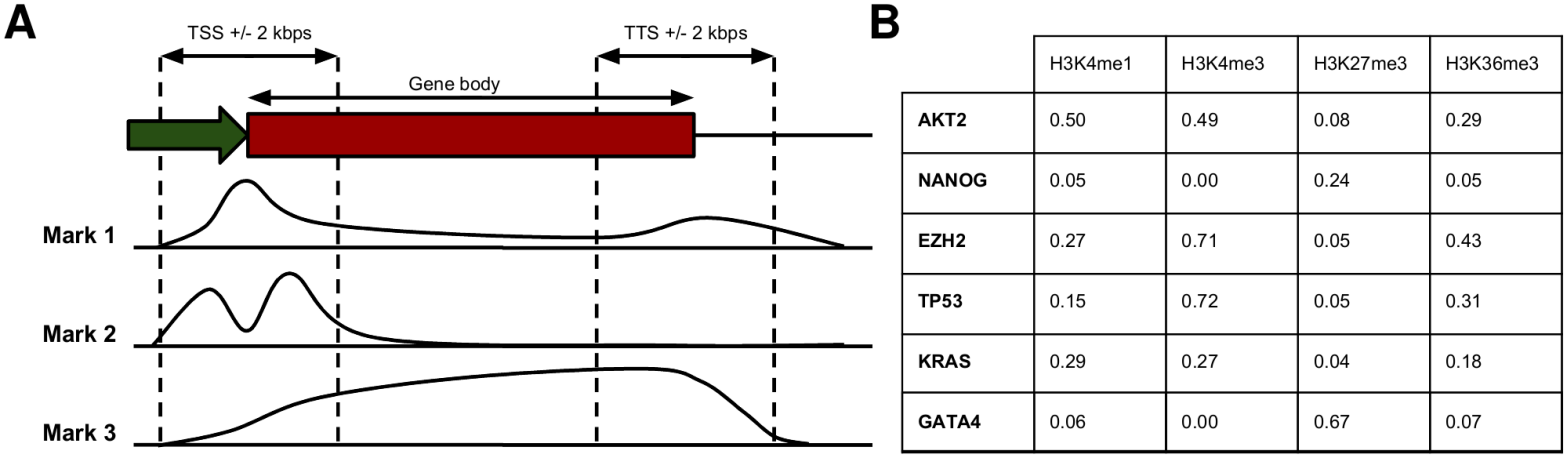
Data processing. (**A**) For each protein coding or lincRNA gene we consider the +/− 2kb region around the outmost TSS (left two vertical dashed lines), the entire transcript (the “gene body”), and the +/− 2kb region around the outmost TTS (right two vertical dashed lines) and count the number of tags for each mark that fall into each region. (**B**) For each constellation we obtain a matrix, where each entry contains the enrichment of a particular mark at a particular gene (for a given gene region). Here, as an illustrating example an excerpt of the matrix for the region around TSSs of protein coding genes in K562 is shown.

In this study, we analyzed genome-wide data obtained from the ENCODE and NIH Roadmap Epigenomics consortia for five cell lines (GM12878, H1, H9, IMR90, and K562). We selected these five cell lines as GM12878, H1, and K562 are Tier 1 cell lines in the ENCODE consortium, and therefore ChIP-seq data for histone modifications as well as a large number of regulatory proteins were available. Furthermore, in the NIH Roadmap Epigenomics Consortium the largest datasets for histone modifications and DNA methylation data were available for the cell lines H1, H9, and IMR90. In total, data was available for DNA methylation, DNase hypersensitivity, 18 chromatin remodelers, 21 coregulators, 30 histone modifications, and 106 transcription factors in these cell lines (**Table A** in S1 Dataset). Additionally, in these cell lines, ChIP-seq data for 14 proteins with unknown or nonexistent regulatory function were available. This rich dataset allowed us to probe associations between regulatory proteins and many epigenetic marks. For both protein coding and lincRNA genes in each cell line, we took all transcripts (with their respective TSSs and TTSs) and considered three regions (see “Materials and Methods”): +/− 2kbs from the most upstream transcription start site (TSS), +/− 2kbs from the most downstream transcription termination site (TTS), and the entire gene body between the most upstream TSS and the most downstream TTS. Whenever we talk about a “gene type”, we mean either protein coding or lincRNA genes. By “region types” we denote the +/− 2kb region around TSSs, gene bodies or the +/− 2kb region around TTSs. In the following, we will refer to a combination of a specific cell line, gene type, and region type, as a “constellation”. As an example, all TSSs (region type) of protein coding genes (gene type) in K562 would be termed a constellation. When we focus on a specific gene type and region type, we will call this a “locus constellation”, so all TSSs of protein coding genes would be termed a locus constellation. We have divided all regions included in this study into either 1 bin or 40 bins. The latter was applied just to regions around TSSs and is only used for predicting gene expression. We tabulated the count of tags that fall into each bin (divided by bin size) for each type of mark (Fig 1A). For DNA methylation data, we also divided these bin counts by the number of CpGs in the corresponding genomic regions (see “Materials and Methods”). Finally, we set the top 1% of values to 1, the bottom 1% of values to 0, and scaled the remaining values linearly between 0 and 1. This results in a table of enrichment values for each mark at each bin (Fig 1B), which we then used for further analysis. We alternatively created enrichment tables using 5%-quantile cutoffs for normalization. We found that downstream results were independent of the specific normalization method (compare **Table B** with **Table C** in S1 Dataset).

### Prediction of marks from other marks

To predict the enrichment value of one mark using the other marks, we fitted a linear model to the data for all marks for a given constellation using the 1-bin resolution data,

$$\begin{array}{ccc} {\text{mark}}_{i} & ∼ & b_{i}+\sum_{j\neq i}a_{j}{\text{mark}}_{j}\\ & = & b_{i}+a_{1}{\text{mark}}_{1}+\ldots +a_{i-1}{\text{mark}}_{i-1}+a_{i+1}{\text{mark}}_{i+1}+\ldots +a_{n}{\text{mark}}_{n}, \end{array}$$

where *n* is the number of marks, mark_*k*_ is the enrichment of the *k*-th mark, and *a*_1_, …, *a*_*i*−1_, *a*_*i*+1_, …, *a*_*n*_, *b*_*i*_ are constants. We used 10-fold cross-validation (CV) to test the generalizability of our predictions.

Then, for a given constellation, we evaluated the Pearson correlation coefficient (Pearson’s r) between the measured and the predicted enrichment values for each mark.

**Fig 2:**
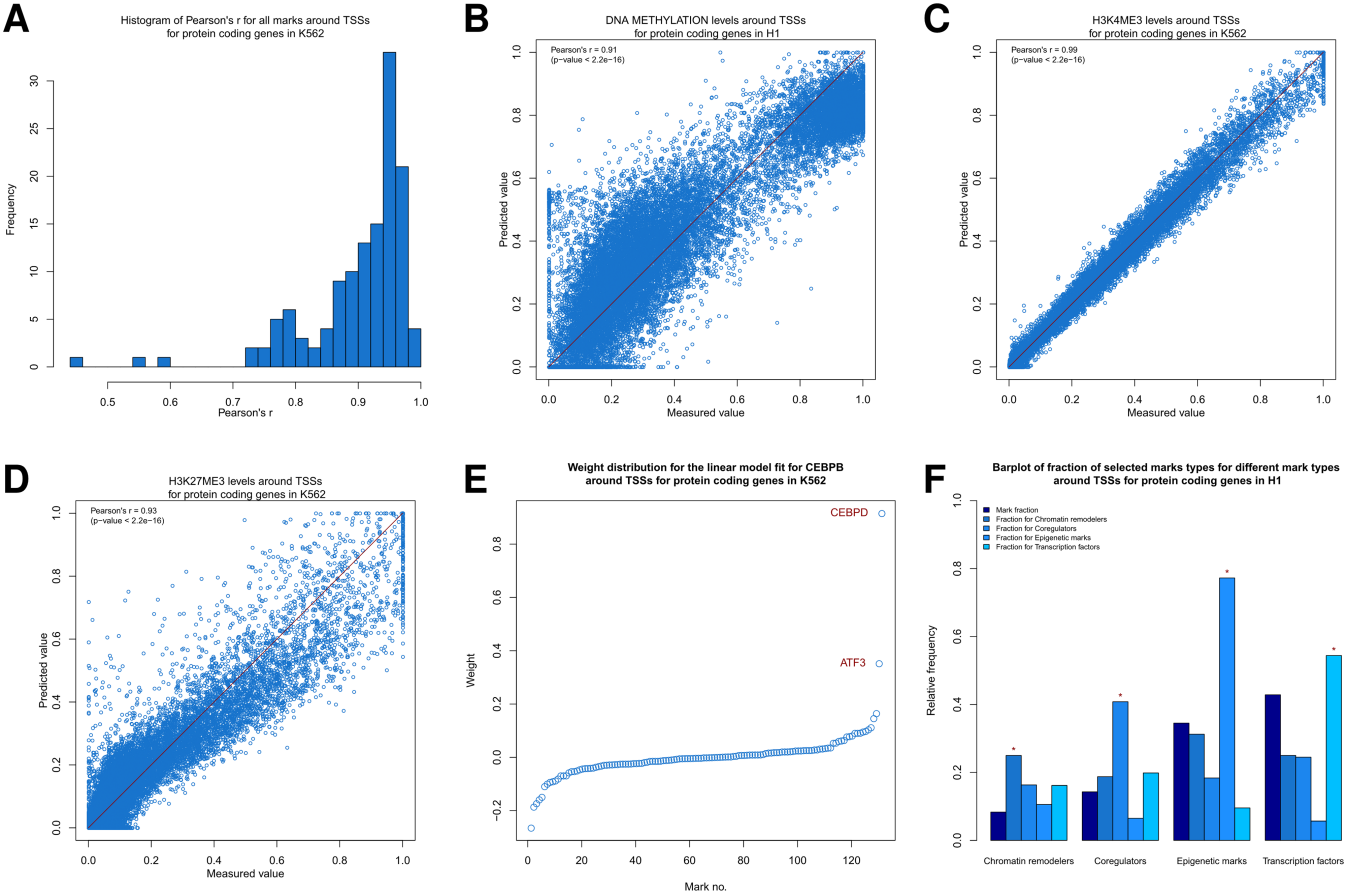
Prediction of a mark by all other marks. (**A**) Histogram of Pearson’s r values between measured and predicted values using 10-fold CV for all marks around the TSSs of protein coding genes in K562 cells. (**B,C,D**) Scatter plot comparing predicted and measured values (10-fold CV) for (**B**) DNA methylation, (**C**) H3K4me3, and (**D**) H3K27me3 around the TSSs of protein coding genes in K562 cells. The line “*y* = *x*” is indicated in red for reference. (**E**) Mark weight distribution in the linear model fitted for *CEBPB* on 100% of the data around TSSs of protein coding genes in K562. (**F**) Barplot of selected mark types for different mark types from the linear models fitted for all marks on 100% of the data for TSSs of protein coding genes in H1. We considered the four different mark types (chromatin remodelers, coregulators, epigenetic marks, and transcription factors) and calculated the relative frequency of each mark type (dark blue bars). Then, for each mark we considered all mark weights of these four types, i.e., without those with an unknown respectively not regulating function. We took a 95% quantile cutoff over all absolute weights, where we considered the weights for all mark models combined. For each mark type, we considered those weights in the linear models for each mark of that respective type, whose absolute weight was above the cutoff, grouped these weights according to the type of the input mark, and plotted the respective relative frequency of each input mark type in the bars of the same color. The bars, where the predicted mark type and the input mark type are identical, are marked with a red star.

The median Pearson’s r was 0.92 over all marks and all possible constellations (S1 Fig and **Table B** in S1 Dataset, p <2.2e-16 each). We conclude that a linear model can predict most marks with great accuracy. This also holds true for specific constellations. As an example, predicting enrichments of marks around the TSSs of protein coding genes in K562 cells, resulted in a median Pearson’s r of 0.94 (Fig 2A and **Table B** in S1 Dataset). In addition, the predicted and the measured values usually are very close to each other (Fig 2B–2D).

In particular, the models for the normalized DNA methylation abundance (Fig 2B) enable us to distinguish between strongly and weakly methylated regions, since most normalized DNA methylation levels close to 0.2 or close to 0.95 have the highest frequency and the minimum frequency occurs around 0.6 (S2 Fig). Further, we can even predict patterns of sequence-specific factors like GATA1, GATA2 or CTCF reasonably well (median Pearson’s r of 0.96, 0.90, and 0.89, respectively, over all constellations for each respective mark). This observation is remarkable given that we are analyzing only the neighborhood of the mark, yet do not include any locus-specific DNA sequence information.

Ernst and Kellis [9] evaluate the performance of their models by comparing the enrichment of a mark against the same mark from other cell lines. Towards this aim, they determine to what degree their models are more accurate than taking a best-performing signal track for the same mark from another cell line respectively the signal average from all samples of the considered mark in other cell lines. When following a similar approach by comparing our models to both the best correlated mark in the same cell line (**Table D** in S1 Dataset) and the enrichments of the same mark from other cell lines (**Table E** in S1 Dataset), for all marks around TSSs at protein coding genes our models outperform the best-correlated mark enrichment in the same cell line (S3A and S3B Fig). Further, for nearly all marks our model performs better than using the same mark enrichments in another cell line (S3C Fig), which holds for all histone modifications (S3D Fig). Similar results can also be observed for other locus constellations (compare **Table B** with **Table D** and **Table E** in S1 Dataset). We conclude that our model evaluation agrees well and compares favorably with the fundamental observations by Ernst and Kellis [9], although, in contrast to them, we do not incorporate information for a specific mark and locus from other cell lines. The complete linear models for all marks and all constellations fitted on all data can be found in the SI (**Table F** in S1 Dataset).

When we assess the weight distribution of the fitted linear models for the individual marks and constellations from above, we observe a weight value close to 0 for most marks and a large values for only a few marks (Fig 2E, S4 and S5 Figs). We suggest this reflects few (functional) interactions among marks, resulting in a sparse interaction network. For instance, in the case of *CEBPB* binding around TSSs of protein coding genes in K562, there are only two marks (out of 131 other marks), that have an absolute weight above 0.3. These are *ATF3* and *CEBPD*, which are both known to interact directly with *CEBPB* [15, 16]. We must caution, however, that higher respectively lower absolute edge weights do not necessarily imply the presence respectively the lack of a biochemical interaction. For instance, *GATA1*, which is an important regulator of erythroid development by regulating large numbers of genes [17], forms a complex with *P300* [18] and is well correlated with it in K562 (**Table D** in S1 Dataset), but the model fitting assigns large absolute weights to other marks for predicting *GATA1* binding to DNA in K562 cells, which apparently possess a similar or better information content than *P300* in this context (**Table D** and **Table F** in S1 Dataset).

Next, we searched for mark types (chromatin remodelers, coregulators, transcription factors, and epigenetic marks like histone modifications, DNA methylation, and DNase hypersensitivity) with significant overrepresentation among the strong model weights for the target mark types. Interestingly, in the case of TSSs of protein coding genes in H1, we do see for all mark types that in the models the identical mark types are overrepresented regarding the relative frequency of that mark type (red stars in Fig 2F). For epigenetic marks, this observation is consistent for all other constellations. Conversely, transcription factors are mostly underrepresented for epigenetic marks (S6 and S7 Figs). Taken together, from an information content point of view our observations suggest that strong links between epigenetic marks appear to exist. This leads us to hypothesize that histone mark patterns are established in a highly coordinated fashion, e.g. if one particular histone modification is set at a position, a defined and characteristic set of histone modifications will be present or absent.

### Activating mark models are generally applicable and silencing mark models are cell line-specific

We have shown that our linear models are capable to faithfully recapitulate relationships between different marks in a particular constellation. We tried to expand on this observation by asking which of our models generalize across the diverse cell lines in our dataset. For this, we took for each ordered pair of different cell lines all marks available in both cell lines, fitted a linear model for each mark and each locus constellation in the first cell line on 100% of the loci and predicted the measured values in the second cell line (see “Materials and Methods”).

**Fig 3:**
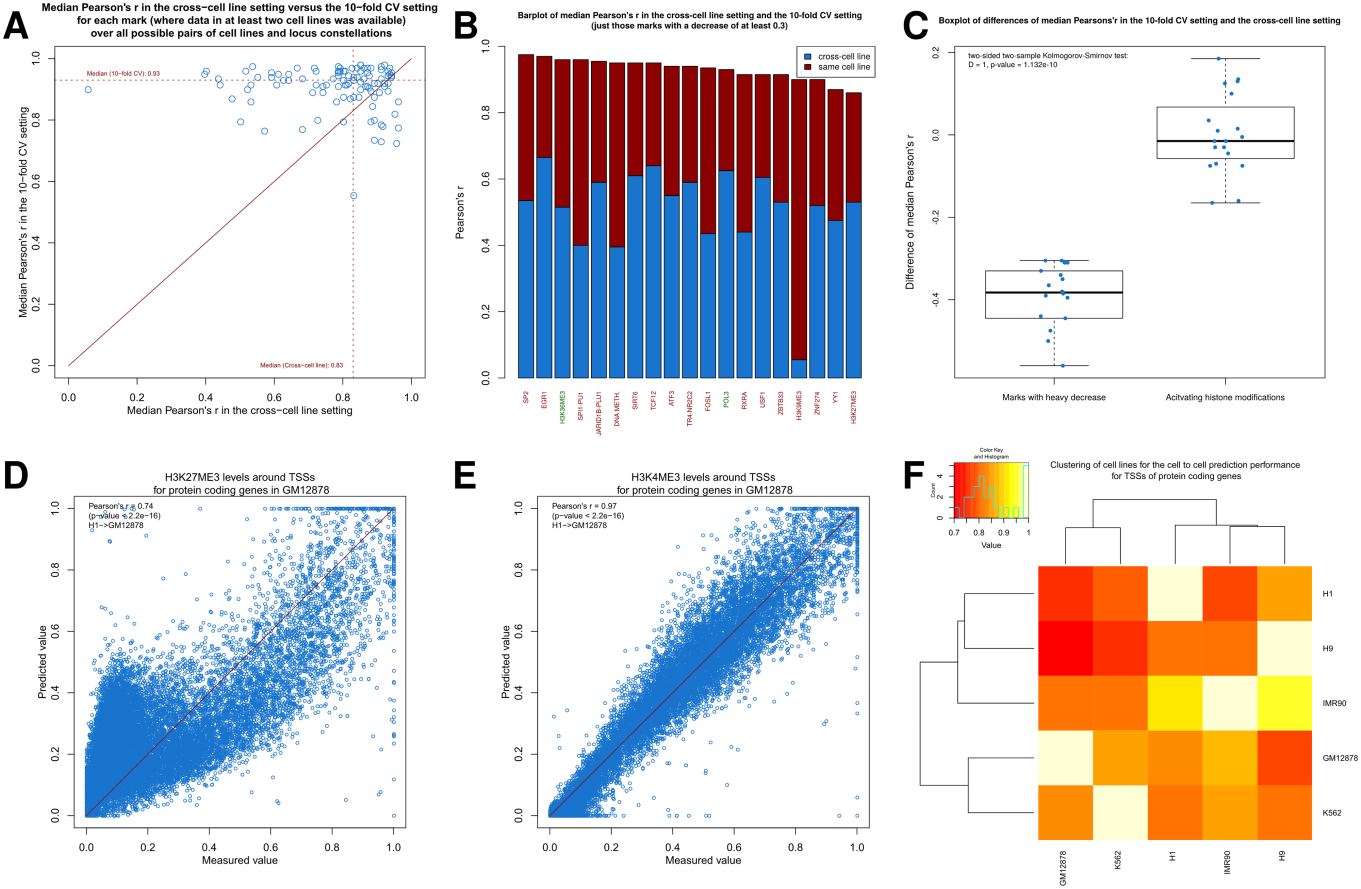
Prediction of a mark by cross-cell line models. (**A**) Scatter plot for the Pearson’s r comparison for each mark (with data for at least two cell lines) between the median correlation between predicted and measured values, when the models, with which the predictions are made, are fitted in other cell lines (on all marks that are present in both cell lines), and the intracellular models. For the first part for each respective mark (with data for at least two cell lines), for each locus constellation, and all ordered pairs of different cell lines, where data for the mark of interest is available for both cell lines, we fit a model for that mark on all other epigenetic marks, for which data is available for both cell lines, in the first cell line, predict the enrichment of epigenetic mark of interest in the second cell line, calculate the Pearson’s r between predicted and measured values, and take the median over all these values for that mark. For the second part for each respective mark (with data for at least two cell lines), we take the median over all 10-fold CV Pearson’s r values of the intracellular models between predicted and measured data for that mark over each locus constellation and cell line, where there is data for that mark available. The median over these median values is shown as dashed red lines. The solid line “*y* = *x*” is indicated in red for reference. (**B**) Barplot of median Pearson’s r in the cross-cell line setting and the 10-fold CV setting, where we displayed just those marks with a decrease of at least 0.3. Marks labeled in red are known to have a silencing function and the marks labeled in green are positively associated with gene expression. (**C**) Boxplot of difference between median Pearson’s r in the cross-cell line setting and the 10-fold CV setting for those marks with a decrease of at least 0.3 (left panel) and activating histone modifications, which means here all histone acetylations and H3K4 methylations (right panel). (Two-sided two-sample Kolmogorov-Smirnov test: D = 1, P = 1.132e-10) (**D,E**) Scatter plots between predicted and measured values for (**D**) H3K27me3 and (**E**) H3K4me3 for TSSs of protein coding genes in GM12878 cells, when the model was fitted in H1. The line “*y* = *x*” is indicated in red in the scatter plots for reference. (**F**) Heatmap showing median Pearson’s r between predicted and measured values for TSSs of protein coding genes over all marks in that target cell line, that are also present in the starting cell line, where the models for the prediction are fitted in the starting cell line and then used to predict the enrichments in the target cell line. For each entry, the target cell line is named as the row entry and the starting cell line is named as the column entry.

When passing from the intracellular 10-fold CV to the cross-cell line setting, we do see on average a slight performance decrease for each mark (Fig 3A). The median Pearson’s r was 0.83 over all marks, all ordered pairs of different cell lines, and all locus constellations (**Table G** in S1 Dataset, p-value of t-test <2.2e-16 for 3893 of the 3948 Pearson’s r values). This finding suggests that the majority of marks can be predicted with an acceptable performance by other marks with rules that hold generally.

There are 19 marks standing out, because their median Pearson’s r fell by more than 0.3 compared to the median Pearson’s r in the above intracellular 10-fold CV setting for each mark (Fig 3B). 17 of the 19 marks are known to represent repressive marks or marks, which have been reported to play a role in gene silencing mechanisms: DNA methylation, H3K9me3, H3K27me3, *ATF3* [19], the histone demethylase *KDM5B* [20] (also known as *JARID1B* and *PLU1*), the histone deacetylase *SIRT6* [21], *TCF12* [22], *USF1* [23, 24], *NR2C2* [25–27] (also known as *TR4*), *YY1* [28, 29], *FOSL1* [30, 31], *SP2* [32, 33], *ZBTB33* [34, 35], *ZNF274* [36, 37], *EGR1* [38, 39],*RXRA* [40], and *SPI1* [41–43] (also known as *PU1*). We note that some of these marks, like *YY1* and *SPI1*, are also known to play a role in gene-activating mechanisms. The remaining two mark H3K36me3 and *POL3* are positively associated with gene expression.

Most notably though, the prototypic silencing marks (DNA methylation, H3K9me3, and H3K27me3) are all included in this set. That is particularly interesting for DNA methylation: In our analysis, it is overall a rather invariable mark between different cell lines (**Table E** in S1 Dataset) and it can be accurately predicted by other marks in each cell line (**Table B** in S1 Dataset). However, the cross-cell line performance of our models is significantly diminished, suggesting that DNA methylation seems to interact with a highly cell type-specific set of marks, while not changing much between different cell lines. Thus one could speculate that DNA methylation serves mostly as a recruiter of marks in a cell type-specific manner.

An alternative explanation, however, could be that this drop in performance is explained by technical variability in mapping epigenetic marks. Therefore, we evaluated this alternative hypothesis in the case of H3K27me3 by fitting the models of H3K27me3 in each cell line, comparing the predictions with an independently generated data track for H3K27me3 in the same cell line, and evaluating the drop in performance. Here the median reduction of the Pearson’s r was just 0.01, so the possibility of technical reasons for the aforementioned drop in performance is unlikely (data not shown).

In contrast to the silencing epigenetic marks, the activating histone modifications, e.g., histone acetylations and H3K4 methylations, had a median drop of 0.01 (Fig 3C). An illustrating case can be seen for H3K27me3 and H3K4me3, when the models are fitted in H1 and evaluated in GM12878 (Fig 3D and 3E).

Our findings suggest that activating marks follow association rules that hold throughout various cell lines and possibly interact with other marks in the very same manner, whereas silencing marks follow more cell line-specific rules and might possess unique interaction partners in each cell line. This finding further suggests that proteins mediating or interacting with activating marks are more ubiquitously expressed and active at similar levels across many cell types and that those proteins that mediate or interact with silencing marks might vary substantially from cell type to cell type in their expression and activity patterns [44].

When comparing the cross-cell line linear model performance with the performance of taking data for the identical mark from another cell line as prediction, we observe that the cross-cell line model performance is better for most marks (S8A Fig) and better for all but three histone modifications (S8B Fig) around the TSSs of protein coding genes. This behavior is similar for other locus constellations (**Table E** and **G** in S1 Dataset). These results are good agreement with the findings made by Ernst and Kellis [9].

Our model comparisons across cell types enable the clustering of samples by using the predictive strengths as a distance metric. When we fit a model for a specific mark in cell line 1 in order to evaluate it in cell line 2, we can cluster the cell lines with regard to how well one cell line predicts the mark enrichments of another one (Fig 3F). Here we see for TSSs of protein coding genes that, as expected, the embryonic stem cells H1 and H9 cluster together as do GM12878 and K562, both being hematopoietic cell lines. Other loci constellations show these groupings consistently as well (S9 Fig). These observations point towards the conclusion that cell lines of similar origin do have more similar association rules for all marks. The empirical evidence is limited though, as we focused only on comparatively few cell lines and because the clustering of cell lines might be biased by the set of marks, for which data is available. This issue will be addressed below (cf. section “Prediction of marks from IHEC histone modifications”).

### Prediction of gene expression

When modeling gene expression (GEx, see “Materials and Methods”) for both protein coding and lincRNA genes, based on thermodynamic principles an exponential relationship between gene expression and epigenetic marks, TFs, and coregulators has been suggested [45, 46]. To further explore this concept within our study, we fitted the linear model for a fixed cell line and a fixed gene type

$$\begin{array}{c} gex∼b+\sum_{i=1}^{n}\sum_{bin=1}^{m}a_{i,bin}{\text{mark}}_{i,bin}. \end{array}$$ (1)

where *gex* is obtained by taking log(GEx + *ε*), setting the top 1% of values to 1, the bottom 1% of values to 0 and then scaling the rest linearly between 0 and 1, *n* is the number marks, *m* is the number of bins (either 1 or 40), mark_*i,bin*_ stands for the enrichment value of the *i*-th mark at the respective bin around the TSS, and *ε* is a small pseudocount accounting for genes with GEx values of 0. At 40-bin resolution, we considered a third alternative, where we only took data of the middle two bins for each mark (bin 20 and 21) of the 40 bins into account. In that case, we restricted our model to the information in the +/− 100 bp region around the TSS.

Since it could be, that a mark has an impact on gene expression only beyond a certain binding strength/likelihood, we also fitted, in addition to linear models, multivariate adaptive regression spline (MARS) models [47], which can account for such effects. These are of the form

$$\begin{array}{c} gex∼c_{0}+\sum_{i=1}^{n}\sum_{bin=1}^{m}\sum_{j=1}^{k_{i,bin}}c_{i,bin,j}B_{i,bin,j}\left({\text{mark}}_{i,bin}\right), \end{array}$$ (2)

where each *B_i,bin,j_*(mark_*i,bin*_) is a piecewise linear function (see “Materials and Methods” for further details).

**Fig 4:**
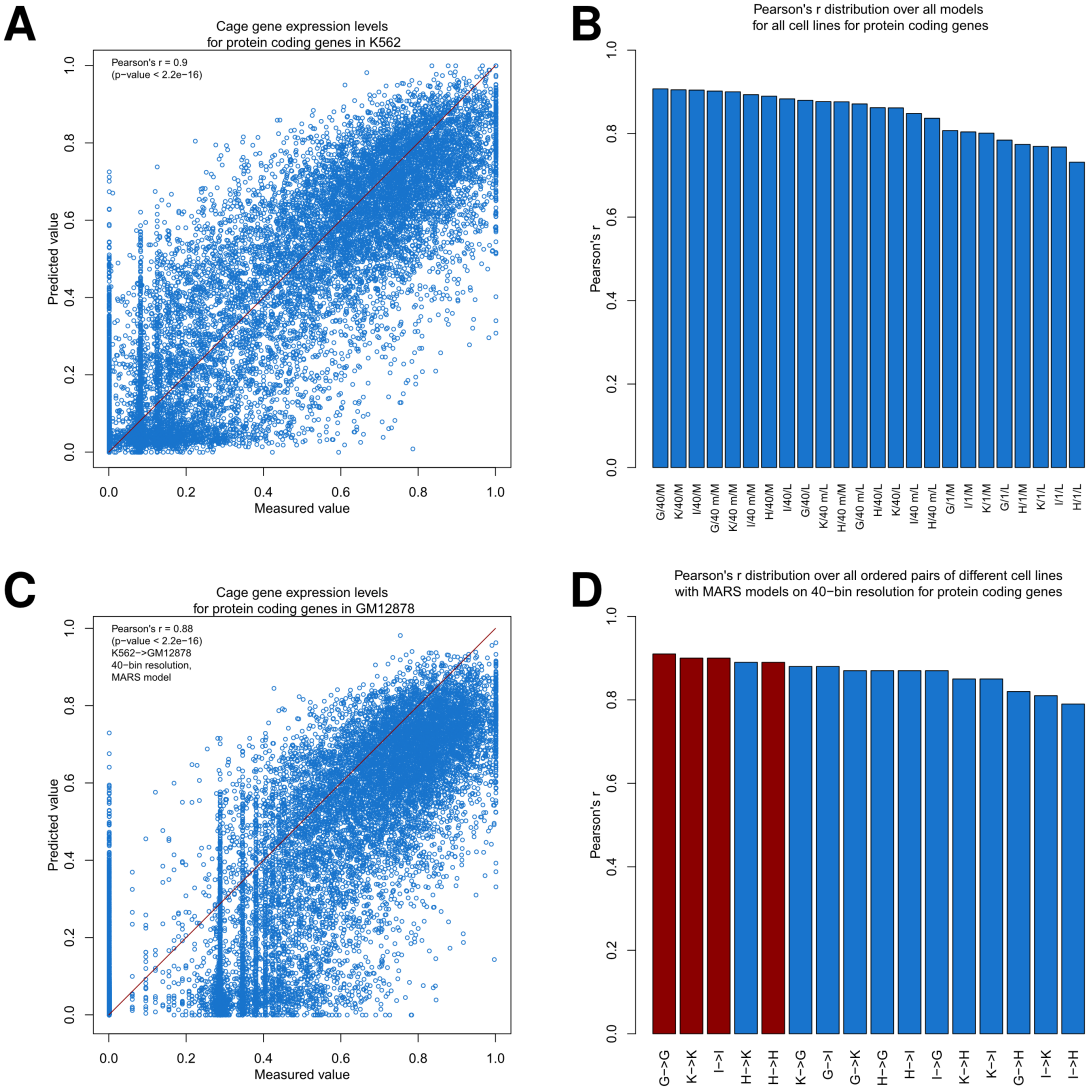
Predicting CAGE gene expression. (**A**) Scatter plot between predicted and measured values (when using 10-fold CV) for CAGE gene expression for protein coding genes in K562 cells when 40-bin resolution data was taken for the input marks of the MARS model (pseudocount *ε* optimized). (**B**) Barplot of Pearson’s r (when using 10-fold CV) for different models for protein coding genes. The bar labels are encoded by their model index, where the first letter represents the cell line (K = K562, G = GM12878, H = H1, I = IMR90), the middle symbols stands for the data input (1 = 1-bin resolution, 40 = 40-bin resolution, 40 m = middle two bins for each mark in 40-bin resolution), and the latter represents the model type (L = linear model, M = MARS model). For each of these the pseudocount *ε* was optimized. (**C**) Scatter plot between predicted and measured values for CAGE gene expression for the protein coding genes in GM12878, when a MARS model on 40-bin resolution data was fitted in K562 cells. The pseudocount *ε* is the same for calculating the logarithmized gene expression in both cell lines by using the optimized *ε* for K562 cells in the 10-fold CV setting. (**D**) Barplot of Pearson’s r values for protein coding genes, when considering each possible ordered pair of different cell lines (analogous to (**C**), with labels as in (**B**)), shown in blue, and the Pearson’s r (when using 10-fold CV) for individual cell lines, shown in red, when using MARS models with 40-bin resolution.

We then used 10-fold CV for both linear and MARS models, 1-bin and 40-bin resolution (all bins and middle two bins) around TSSs, and varying pseudocounts (**Table H** in S1 Dataset). When using the best model for protein coding gene expression we achieved Pearson’s r between predicted vs. measured values of 0.9 for K562 (Fig 4A), 0.91 for GM12878, 0.89 for H1, and 0.9 for IMR90 (Fig 4B, p-value <2.2e-16 each), hence we obtained a similar performance as in Dong et al. [13], although our approach is more straightforward, simpler in its assumptions, and uses fewer bins in and around the genes. Unsurprisingly, in each instance the best performing model was a MARS model (2) on 40-bin resolution (taking all bins into account), though the pseudocounts varied. The top performance of linear models (1) was only slightly reduced (Pearson’s r of 0.89 for K562, 0.88 for GM12878, 0.86 for H1, and 0.88 for IMR90, p-value <2.2e-16 each), as was the case when taking all models on 40-bin resolution, where just the middle two bins were considered (Pearson’s r of 0.9 for K562, 0.9 for GM12878, 0.88 for H1, and 0.89 for IMR90, p-value <2.2e-16 each) or even just taking linear models on the middle two bins (Pearson’s r of 0.89 for K562, 0.88 for GM12878, 0.85 for H1, and 0.86 for IMR90, p-value <2.2e-16 each). However, when we considered just models on 1-bin resolution, we obtained significantly reduced performances (Pearson’s r of 0.8 for K562, 0.81 for GM12878, 0.77 for H1, and 0.8 for IMR90, p-value <2.2e-16 each). Still, these models were in the same performance range as the models used by Karlić et al. [14], which worked with the same resolution.

Just as for the protein coding genes, for lincRNA genes the MARS models on 40-bin resolution are the best-performing ones, where for H1 just the middle two bins are considered (S10 Fig). However, the performance is significantly reduced compared to the performance on protein coding genes (Pearson’s r of 0.79 for K562, 0.78 for GM12878, 0.78 for H1, and 0.77 for IMR90, p-value <2.2e-16 each). The relative drop in performance between protein coding genes and lincRNA genes was similar for both linear and non-linear models. Also, for both modeling approaches for the 40-bin setting case (by considering all 40 bins for all marks or the middle two bins for all marks) the model performance was best, whereas the model performance significantly decreased when applying 1-bin data only.

We conclude from these parameter scans that strong model performances (particularly for protein coding genes) can be achieved with linear or mixed linear models (like MARS) as long as the resolution around the TSS is sufficiently high. Also, the information in the +/− 100 bp region around the TSS seems to be of particular importance for each model’s performance. This conclusion is supported by the case, where we fitted the MARS models on 100% of the data on 40-bin resolution (**Table I** in S1 Dataset), and where primarily mark enrichments either in or close to the +/− 100 bp region are used. A notable exception is H3K36me3, where in 5 out of the 8 displayed models bin positions downstream more than 1500 bps of the TSS were considered. This, however, is in good agreement with the known behavior of H3K36me3 since it accumulates in actively transcribed genes in downstream regions of the gene body.

After we investigated the performance in the intra-cell line setting using 10-fold CV, we tested how cell line-specific or unspecific the models are for protein coding and lincRNA gene expression. Just as in the case of (epigenetic) marks, for each ordered pair of different cell lines we considered the marks for which data is available in both cell lines, fitted a model in the first cell line for either protein coding or lincRNA genes and then predicted gene expression in the other cell line. The performance of these cross-cell line models was comparable to the performance of the models obtained, when using 10-fold CV in the intra-cell line setting (Fig 4C and 4D, S11 Fig, and **Table J** in S1 Dataset). Thus, we obtain that for both protein coding and lincRNA genes the gene expression models, as functions of the marks around the TSS, appear to be independent of the specific cell line.

### Prediction of marks from IHEC histone modifications

**Fig 5:**
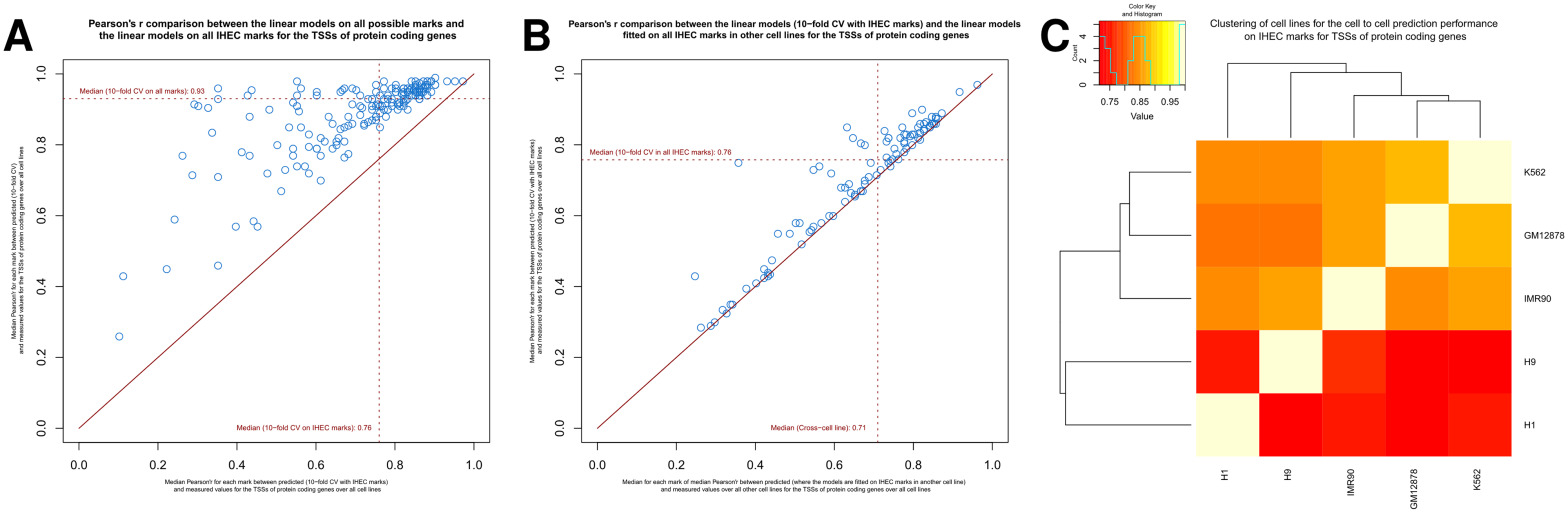
Prediction of a mark by IHEC marks. (**A**) Scatter plot for median Pearson’s r comparison for each mark (apart from the six IHEC histone modifications) at TSSs of protein coding genes between the 10-fold CV model performance, where the models are fitted on all other marks, and the 10-fold CV model performance, where the models are fitted on IHEC marks. The medians over the median Pearson’s r values are shown as dashed red lines. The solid line “*y* = *x*” is indicated in red for reference. (**B**) Scatter plot for median Pearson’s r comparison for each mark (apart from the six IHEC histone modifications), where there is data for at least two cell lines available, at TSSs of protein coding genes between the median 10-fold CV model performance, where the models are fitted on IHEC marks, and the median correlation between predicted and measured values, when the models, with which the predictions are made, are fitted in other cell lines on the IHEC marks. The medians over the median Pearson’s r values are shown as dashed red lines. The solid line “*y* = *x*” is indicated in red for reference. (**C**) Heatmap showing median Pearson’s r between predicted and measured values for TSSs of protein coding genes over all marks for which data is available in all cell lines apart from the IHEC marks (i.e., DNase hypersensitivity, H2.AZ, H3K4me2, H3K9ac, H3K79me2, H4K20me1), where the models for the prediction are fitted on the IHEC marks in the starting cell line and then used to predict the enrichments in the target cell line. For each entry, the target cell line is named as the row entry and the starting cell line is named as the column entry.

One main objective of the IHEC consortium was to create genome-wide, comprehensive maps for six standard histone modifications (H3K4me1, H3K4me3, H3K9me3, H3K27ac, H3K27me3, H3K36me3) complemented by DNA methylation and RNA-Seq data for a large panel of cell lines. This raises the question in how far we can account for the information content of all marks in various cell types by the six IHEC histone modifications. For the sake of simplicity, we will refer to these six histone modifications here as the “IHEC marks”. First, we aimed to predict for all cell lines all other marks from the IHEC marks using linear models at 1-bin resolution. When performing 10-fold CV on each fixed constellation, we obtain a median over each other mark’s median Pearson’s r over all possible constellations of 0.76 (Fig 5A and **Table K** in S1 Dataset, p-value of t-test <2.2e-16 each). When we restrict ourselves to certain locus constellations like the region around the TSS of protein coding genes, the median Pearson’s r over all other marks does not differ (0.76) from the overall median Pearson’s r (S12A Fig). However, when we only consider histone modifications, the median Pearson’s r increases from 0.76 to 0.85 (S12B Fig) and decreases to 0.73 when we consider all other marks except histone modifications (S12C Fig).

If we compare the performance of these reduced models to the performance of the comprehensive models involving all possible marks as above, we see that the results of the latter are significantly better (Fig 5A, the BIC value for the all-mark models is always smaller for all other marks and all cell lines, data not shown), but the difference is smaller for histone modifications (S13A Fig) at TSSs of protein coding genes. This suggests that the IHEC mark subset is recapturing the possible prediction performance of histone modifications by all other marks better than for non-histone marks. Furthermore, the models focusing on the IHEC marks show a better or equally good performance compared to the median correlation of the same mark in another cell line for most marks and for all histone modifications (S13B and S13C Fig) at TSSs of protein coding genes. Hence, for most other surveyed marks, in particular all other histone modifications, the models fitted on the IHEC marks give us a better or equally good prediction performance for TSSs of protein coding genes than taking the enrichments from other cell lines at the respective TSSs. Similar observations can be made at other locus constellations (**Table B**, **E**, and **K** in S1 Dataset).

Next, we assessed the cell line specificity of these models by considering all ordered pairs of different cell lines. We fitted a linear model on the IHEC marks for each other mark available in both cell lines for each fixed locus constellation in the first cell line and used these models to predict the measured values in the second cell line. The median over each possible mark’s median Pearson’s r over all ordered pairs of different cell lines and all locus constellations was 0.71 (**Table L** in S1 Dataset, p-value of t-test <2.2e-16 for 3219 of the 3228 Pearson’s r values). Hence, most of the models perform similarly compared to the 10-fold CV performance in one cell line on the IHEC data (Fig 5B and (S14A Fig) for TSSs of protein coding genes). Additionally, when comparing the cross-cell line IHEC model performance with the performance of taking data for the identical mark from another cell line, we observe that our cross-cell line IHEC model performance is better for the majority of marks (S14B Fig) and better for all but one histone modification (S14C Fig) around the TSSs of protein coding genes, which appears to be a consistent pattern at other locus constellations as well (**Table E** and **L** in S1 Dataset). On the other hand, the cross-cell line model performance, where all marks are allowed, is stronger than the cross-cell line IHEC model performance for both all marks (S14D Fig) and histone modifications (S14E Fig).

The models created for each constellation and all other marks aside from the six IHEC histone modifications on 100% of the data (i.e. we take the IHEC histone modifications at 100% of the loci for that particular constellation as input and fit a model for a certain mark at said loci) can in principle be applied to extend the epigenome and regulatory protein data for any cell line for which ChIP-Seq data for the six IHEC histone modifications are available (**Table M** in S1 Dataset). This is particularly true for marks whose models have been shown to be cell line-unspecific here.

As above (Fig 3F), we tried to cluster cell lines with respect to how well one cell line predicts the mark enrichments of another one with the cross-cell line IHEC models (Fig 5C at TSSs of protein coding genes). Here we restricted the clustering analysis to those six epigenetic marks (DNase hypersensitivity, H2.AZ, H3K4me2, H3K9ac, H3K79me2, H4K20me1) for which data is available in all five cell lines. When focusing on how well their marks are predicted by other cell line models, we observe for loci associated with protein coding genes (Fig 5C and S15A and S15B Fig), that similarly to Fig 3F the two embryonic stem cells H1 and H9 cluster together as do the blood-related cell lines GM12878 and K562. Based on these observations, we conclude that cell lines of similar phenotype show a similar performance for the prediction of enrichments of marks, at least at protein coding genes. It is counterintuitive, however, that the models fitted on embryonic stem cell lines (H1 and H9) are better at predicting the enrichments of the non-embryonic cell lines (GM12878, IMR90, K562) compared to how models fitted on H1 perform on H9 data and vice versa (Fig 5C and S15 Fig).

### Recursive selection of marks according to their information content

Next, we wanted to find out if we can identify an “optimal” subset of marks for predicting many of the remaining marks of a given sample. Towards this aim, we analyzed the information content of the marks by recursively adding them as model input (see “Materials and Methods”). For simplicity, we restricted our calculations to the regions around TSSs of protein coding genes for all cell lines. For each round and cell line, we selected the mark that had the highest median Person’s r over all not yet selected marks through 10-fold CV, when creating linear models with the already selected marks and the current mark as input. The selected mark order differed across cell lines (**Table N** in S1 Dataset). For instance, H3K4me3 is a top mark in four cell lines, but occupies the lowest rank of all 30 marks analyzed in H9. On the other hand, H3K4me2 is strongly correlated to H3K4me3, thus having a similar information content, and is a top mark in H9, but selected only at later stages in the other four cell lines. This is the case, because for our selection method one of these two marks is sufficient to be included at early stages, whereas using both together would not strongly enhance the prediction performance of the selected marks.

**Fig 6:**
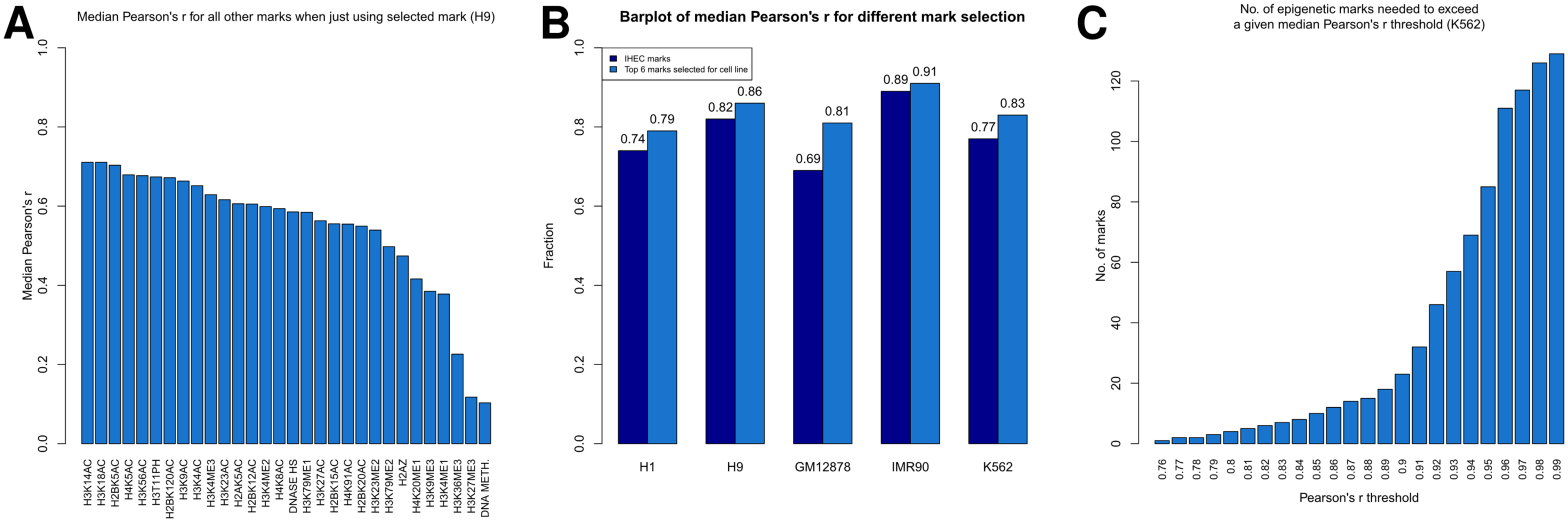
Compression of information content. (**A**) Barplot of median Pearson’s r for each mark comparing the measured and predicted values for all other marks in the region around TSSs of protein coding genes in H9 cells. For each mark, we predicted for each other mark the enrichments using 10-fold CV and fitted linear models with solely the given mark as input (plus constant). Then we calculated the median Pearson’s r between predicted and measured values for all these other marks. (**B**) Median Pearson’s r performance on all other marks when using 10-fold CV and the IHEC marks or the top 6 selected marks for each respective cell line. (**C**) Number of marks that are needed to exceed a given median Pearson’s r threshold in K562 cells.

Unsurprisingly, the rank order of marks is more consistent across cell lines when we rank in each cell line each mark by its median Pearson’s r when it alone, i.e., the models in the first selection round, is used in the linear models to predict the other marks (**Table O** in S1 Dataset). For instance, ChIP-Seq data for the nuclear receptor corepressor/HDAC3 complex subunit *TBLR1* [48] were available only in the cell lines K562 and GM12878, but in both cases it was always selected as the first mark. The median Pearson’s r for predicting all other marks in K562 by *TBLR1* is 0.77 (**Tables P** and **Q** in S1 Dataset), thus having the same information content as the six IHEC histone modifications (median Pearson’s r 0.77). For GM12878, the median Pearson’s r for predicting all other marks by *TBLR1* is 0.71 (**Tables R** and **S** in S1 Dataset), thus having even a stronger median Pearson’s r performance than the six IHEC histone modifications together (median Pearson’s r 0.69). This shows that at least for hematopoietic cell lines analyzed at TSSs of protein coding genes, *TBLR1* has a large information content for all other marks. Also, H3K14ac ranks in the top 6 marks in all cell lines, where data is available, just as GTF2F1 is in the top 4 marks. In contrast, with our analytical approach silencing marks like DNA methylation, H3K27me3, and H3K9me3, provide relatively low predictive value for all other epigenetic marks and regulatory proteins (Fig 6A and S16 Fig).

Generally, when selecting increasingly up to six marks for each cell line we see a significantly enhanced median Pearson’s r for all cell lines compared to the six IHEC histone modifications (Fig 6B), rising from 0.77 to 0.83 in K562, from 0.69 to 0.81 in GM12878, from 0.74 to 0.79 in H1, from 0.89 to 0.91 in IMR90, and from 0.82 to 0.86 in H9 (**Tables P,Q,R,S,T,U,V,W,X**, and **Y** in S1 Dataset). When we start with just one-mark models and then start adding other marks we observe a strong increase in the median Pearson’s r (Fig 6C and S17 Fig). However, after this strong, initial increase the value of adding more marks plateaus and including even more marks only slightly increases the median Pearson’s r. We find the general rule, that selecting few, informative marks can result in a predictive performance of up to a median Pearson’s of 0.9. To improve the predictive values of our models beyond that level, experimental data for a significantly larger number of marks is required. In addition, we have to caution, that a high median value, does not guarantee a good prediction performance for all other marks. For instance, REST never has a Pearson’s r of above 0.35 in this above selection regime for K562 until it is selected as the 94th mark (**Table P** in S1 Dataset).

## Discussion

Eukaryotic gene regulation is characterized by DNA microstates composed of TFs, coregulatory complexes, nucleosomes, histone modifications, DNA methylation, and parts of the three-dimensional architecture of genomes. To resolve the microstates’ apparent complexity could enable fundamental insights into the mechanistic underpinning of the epigenetic regulation of mammalian transcription. Modern whole-genome sequencing methods are now providing a large amount of data for the in-depth analysis of these marks. Since it is known, that many epigenetic marks and regulatory protein colocalize, it is not surprising, that only a small fraction of the potential combinatorial complexity is observable in genome-wide ChIP-seq datasets stemming from the same cell types. However, it has been challenging to chart the combinatorial code of localized signals from epigenetic marks and TFs bound to DNA in a systematic and quantitative way.

In this study, we showed that a small number of marks combined with linear models of low complexity can effectively predict other marks across the genome, although, as a word of caution, this does not necessarily apply to every locus, but usually the vast majority of them. This performance observation holds true both for predictions within individual and across different cell lines. These generalizable “rules of association” encoded in these models were found to be largely cell line-independent for activating marks, but more cell line-specific for silencing marks, for both protein coding and lincRNA genes. Based on these observations, one could speculate if silencing marks may interact with varying binding partners in different cell lines, while activating marks may not.

Linear models are also capable of predicting gene expression levels from marks with high average correlation across the genome. We found that the resolution of data around the transcription start site (TSS) was more important for predicting gene expression than the window width around the TSS. The best-performing models used a 100 bp resolution around the TSS. Utilizing this binning our models with a large window (+/− 2 kb relative to the TSS) and with a relatively small window of only a fraction of the larger window (+/−100 bp) performed similarly well. This observation is suggestive of strong enrichments of predictive marks located very close to the TSS.

The extent of information “compression” that can be achieved depends strongly on the type of marks we included in our analysis. For example, the transducin beta-like 1 X-linked receptor 1 (*TBLR1*), for which we only had data in the two hematopoietic cell lines K562 and GM12878, was always the best mark for predicting all other marks, with a performance equal or superior to models, which used all six of the IHEC histone modifications. Thus, it could prove valuable to advance the study of this nuclear receptor corepressor further [49]. In contrast, silencing marks like DNA methylation, H3K27me3 or H3K9me3 do seem to have much less predictive power compared to other epigenetic or regulatory protein marks. Overall, for each cell line, a relatively modest number of marks, compared to the total measured, were required to predict most other marks with a median Pearson’s r up to 0.9. Improving the model performances beyond this level required the inclusion of a significantly larger number of marks. Of course, it would be desirable to have an optimum number of core marks for imputing other marks. This, however, appears to be very much dependent on the kind of marks we are interested in. For instance, in case we want to study the pattern of activating marks, a handful of histone acetylations might be very informative. If we are interested in other kinds of marks, we might benefit more from a different set of marks.

The ENCODE and NIH Roadmap Epigenomics Consortia have focused on steady-state data for several cell lines, which were chosen because they are considered to be representative for distinct cell types. Based on this type of data, we found a surprisingly strong correlation structure between various marks, indicative of a great information redundancy among different (epigenetic) marks. From another perspective, this could be utilized for predicting most other marks based on few measured signals. We speculate that the dynamic causal relationships between different marks—which marks recruit other marks—and the network effects through which different genes influence each other, will be more difficult to dissect. As we have previously shown this dynamic type of information is required to predict gene regulatory functions [1]. Approaches based on steady-state data [4–6] mostly yield acyclic causal graphs, but are unable to define dynamic rules. As a logical next step, it will be highly instructive to investigate time-resolved ChIP-Seq data at larger scale [50–56] derived from, e.g., cells responding to external stimuli. Ultimately, it will be intriguing to see if the steady-state redundancies identified in this study could be extended towards the highly dynamic mechanisms underlying gene regulation.

## Materials and methods

### URLs

- Encyclopedia of DNA Elements (ENCODE) Consortium, http://genome.ucsc.edu/ENCODE/ (old) and http://encodeproject.org/ (new)
- ENCODE blacklist, http://hgdownload.cse.ucsc.edu/goldenPath/hg19/encodeDCC/wgEncodeMapability/wgEncodeDacMapabilityConsensusExcludable.bed.gz
- NIH Roadmap Epigenomics project, http://roadmapepigenomics.org
- hg19 data, http://hgdownload.cse.ucsc.edu/goldenPath/hg19/bigZips/
- Gencode v. 18, http://www.gencodegenes.org/releases/18.html
- Python package HTSeq, http://www-huber.embl.de/users/anders/HTSeq/
- Python packge wiggelen, http://pypi.python.org/pypi/wiggelen/
- R software, http://cran.r-project.org/
- R package earth, http://cran.r-project.org/package=earth.

### ENCODE and NIH Roadmap Epigenomics mark data processing

We downloaded the ENCODE and NIH Roadmap Epigenomics data (**Table Z** in S1 Dataset) for the five human cell lines (GM12878, H1, H9, IMR90, and K562). For each mark/cell line constellation, we randomly downloaded one of the data files available. The treatment protocols for the cells and the sample preparation and data generation protocols for the different samples can be found on the homepages of the respective consortium. All these data were either wig or bigwig files, where the latter were converted to bedGraph files. Following this, we processed the data with the Python scripts that we have released and documented at http://vcp.med.harvard.edu/linear-epigenome.html, which make use of the HTSeq and wiggelen packages. First, for each protein coding or lincRNA gene, respectively, we determined with the help of the Gencode annotation set (version 18) where the outmost TSS or TTS of all transcripts of the respective gene lie in the hg19 assembly. Then we considered for each gene three regions:

1. +/− 2kbs around the outmost TSS
2. the gene body (the region between the outmost TSS and TTS)
3. +/− 2kbs around the outmost TTS

Following this, for each cell line and each data file for an individual mark we counted the number of tags falling into the aforementioned regions for each gene, where we considered 1-bin and 40-bin resolutions for each of these regions (40-bin resolution was just considered for TSSs). Then we stored for each resolution type and each region type the result for each gene and each bin, where we divided each count for the bin by the size of the bin. The latter matters only since the gene body varies in length from gene to gene. In addition to that for DNA methylation data we also count the number of CpGs in each of the above regions and bins from the hg19 sequence data and normalize the DNA methylation values regarding the number of CpGs.

After we did this for both protein coding and lincRNA genes, we created with R [57] a matrix for each cell line, region, and bin resolution, where we essentially glued together all of the above output for each mark available for this cell line, only for DNA methylation we took once the absolute value from above and then the normalized value with respect to the number of CpGs in the regions. We deleted those entries for genes, where we have an overlap with the ENCODE blacklist, which includes loci where artifact signals for ChIP-Seq and DNase-Seq data are known, and excluded those genes localized on the sex chromosomes. Following this, we took for each mark the one respectively 40 columns for this mark and set the bottom 1% to 0, the top 1% to 1, and scaled the rest linearly between 0 and 1. Hence, e.g., for the TSS region of protein coding genes in K562 in 1-bin resolution we obtained a matrix of dimension 19399 × 132, where 19399 is the number of genes, 132 is the number of marks, and each entry at position (*i*, *j*) reflects the enrichment of mark *j* at the TSS of gene *i*. If we consider the 40-bin resolution in the same setting, we obtain a matrix of dimension 19399 × (40 ⋅ 132), since for each mark we have 40 columns. If we consider a cell line like H9, where we have DNA methylation data at hand, we obtain in the 1-bin setting for protein coding genes a matrix of dimension 19399 × 31, where we have 30 marks for that cell line, but included two columns for DNA methylation, one for the absolute value and one for the normalized value with respect to the number of CpGs in the region. All generated data can be found at http://vcp.med.harvard.edu/linear-epigenome.html.

### Model fitting, measuring Pearson’s r and p-value

Unless otherwise stated, we used the built-in R function lm() for fitting linear models. For the gene expression analysis, we also used multivariate adaptive regression spline (MARS), where the fitted models are weighted sums of piecewise linear functions *B*(*x*), which are of the form max(0, *x* − *c*) or max(0, *c* − *x*) for some constant *c*. Here we used the R package *earth* and the function earth(). We obtained the Pearson’s r between the measured and predicted values and the p-value of t-test by the built-in R function cor.test().

### Predicting one mark from the other marks

For a fixed gene type (protein coding or lincRNA genes), region type (+/− 2kbs around TSS, gene body or +/− 2kbs around TTS), and cell line we first took one column out of the matrix for this locus constellation, which corresponds to the mark of interest. For DNA methylation, we used the normalized data values (see above). For the remaining marks, we removed the absolute DNA methylation data when predicting DNA methylation. When predicting values other than DNA methylation, although DNA methylation data were available for this cell line, we deleted the normalized DNA methylation data as we were only interested in the total presence of DNA methylation and its effects. We then used 10-fold CV to obtain the predictions. For fitting the 100% models we simply considered 100% of the genes, fitted a model for a given mark and constellation, and extracted all weights from this linear model.

### Predicting the enrichment of one mark with cross-cell line models

For a fixed ordered pair of different cell lines, e.g., (K562,GM12878), and fixed locus constellation, we considered all marks for which data in both cell lines was available. We reduced the respective matrices for both cell lines here to these marks. Once we fixed a mark of interest (for which we do have data in both cell lines), we took this mark out of the matrix for the first cell line and obtained a vector and the remaining matrix (DNA methylation was treated as in the paragraph from above). We fitted a model for this mark in the first cell line on the 100% data and tried to predict the mark in the second cell line by using data for all other marks that were present in both cell lines. We measured the Pearson’s r and the p-value by comparing the measured and predicted value for the mark of interest in the second cell line.

### Processing CAGE gene expression data

Gene expression data was available only for four of the cell lines (K562, GM12878, H1, and IMR90) and we chose to take CAGE nucleus data (**Table Z** in S1 Dataset). We processed these data exactly like the mark data on 40-bin resolution around the TSSs for both gene types (apart from the normalization between 0 and 1). Since we had plus and minus strand data files for each cell line, we simply summed for each gene the middle two bins for both data files, i.e., we define the gene expression for a gene by *GEx* = *bin*_20,+_ + *bin*_21,+_ + *bin*_20,−_ + *bin*_21,−_, where *bin*_*j*,⋆_ should indicate the value of bin *j* for the respective strand file (⋆) for a particular gene of interest. Hence, we took the sum of values in the +/− 100 bp region around the TSS for both strand information data.

### Predicting (CAGE) gene expression

For a fixed cell line of the four cell lines mentioned above and a fixed gene type, we took the gene expression data file from above and added various pseudocounts *ε* = 0.001, 0.01, 0.1, 1 to each gene, respectively, logarithmized the data, set the top 1% of the data to 1, the bottom 1% to 0, and scaled the rest linearly between 0 and 1, which we name *gex*. Then we considered three data inputs: In the first data input, we took 1-bin mark resolution data around the TSSs of this gene type, in the second it was 40-bin resolution data, and in the third it was 40-bin resolution data, where for each mark we just considered the two middle bins as input. If DNA methylation data was available, we just considered the absolute (not the normalized) DNA methylation data. The models were fitted as a (completely) linear model (lm()) or as a (piecewise linear) MARS model (earth()). In analogy to predicting the marks we used 10-fold CV here and calculated the Pearson’s r and the p-value between the measured and predicted values.

### Predicting (CAGE) gene expression with cross-cell line models

For a fixed ordered pair of different cell lines, gene type, input data type for the marks (1-bin resolution, 40-bin resolution or just the middle two bins in 40-bin resolution), and model type (linear or MARS model), we took the *ε* from above that maximized the Pearson’s r for the first cell line for that gene type, input data type, and model type. We obtained the gene expression (*gex*) for the gene type of interest in both cell lines with respect to the *ε* in the first cell line just as above. We took only those marks for the model fitting (in the first cell line) and validating (in the second cell line), that were present in both cell lines, where again only absolute DNA methylation data was taken, if available. We then fitted the model on 100% of the data in the first cell line, predicted the gene expression in the second cell line, and calculated the Pearson’s r and p-value between predicted and measured values.

### Predicting marks from the IHEC histone modifications

For a fixed constellation we took the data from the six IHEC histone modifications (H3K4me1, H3K4me3, H3K9me3, H3K27ac, H3K27me3, H3K36me3) and fitted a model for each other mark available (i.e., aside from these six histone modifications). Again, if we wanted to predict DNA methylation we just considered the normalized value. We used 10-fold CV to obtain the Pearson’s r and p-value. For establishing the models on 100% of the data we did the same analysis as in the case, where all other marks were used to predict a particular mark of interest.

### Predicting the enrichment of marks from the IHEC histone modifications with cross-cell line models

For a fixed ordered pair of different cell lines and fixed locus constellation, we applied the same strategy as above with the only difference being that the input data was restricted to the six IHEC histone modifications. The marks that were to be predicted were marks for which we have data for both cell lines (aside from the six IHEC histone modifications).

### Selecting marks according to their information content

For the sake of simplicity, we focused here on the +/− 2 kb regions around TSSs of protein coding genes. For each cell line (where *n* is the number of marks for the given cell line) we applied the following algorithm:

  // Initialize the set of chosen marks *S*

  *S* → ∅.

  // Initialize the set of all marks *K*

  *K* → {mark_1_, …, mark_*n*_}.

  // Initialize the selection order vector *ordervec*

  $ordervec\to 0\in R^{n}$.

  // *i* indicates the number of the selection round

  For *i* = 1, …, *n*:

    // initialize vector *v* for the median Pearson’s r

    // of the not yet selected marks

    $v\to 0\in R^{n+1-i}$.

    // mark^(*j*)^ loops over all not yet selected marks

    For mark^(*j*)^ ∈ *K*\*S*:

    Set *S*_*j*_ = *S* ∪ {mark^(*j*)^}.

    Predict for each mark in *K*\*S*_*j*_

    the Pearson’s r when using 10-fold CV

    with input marks *S*_*j*_ for the model

    (DNA methylation as usual).

    *v*_*j*_ → median of the Pearson’s r values.

    Let mark_*m*_ correspond to the maximum entry of *v*.

    // Extend *S* by mark_*m*_.

    *S* → *S* ∪ {mark_*m*_}.

    // Set *i*—th entry of *ordervec* to *m*.

    *ordervec*_*i*_ = *m*.

The vector *ordervec* gives us the selection order.

## Supporting information

S1 Dataset Supporting tables. This file contains 27 sheets, where we do have information about the marks used, Pearson’s r and p-values for each situation evaluated, fitted linear models on 100% of the data, mark information content, and a list with download links.
(XLSX)

S1 Fig 10-fold CV model performance histogram. Histogram of Pearson’s r between measured and predicted values (when using 10-fold CV) for all marks and constellations.
(TIF)

S2 Fig Normalized DNA methylation enrichments. Histogram of normalized DNA methylation enrichments in the regions around TSSs of protein coding genes in H1. Here a value of 0 means that 0% of the CpGs are methylated, and a value of 1 means that 100% of the CpGs are methylated.
(TIF)

S3 Fig 10-fold CV model performance comparison against “reference models”, where the “predictions” are the enrichments of other ChIP-seq data. (**A**) Scatter plot for median Pearson’s r comparison for each mark at TSSs of protein coding genes between the 10-fold CV model performance and the correlation of the best correlated mark in the same respective cell line. That means for each mark we take the median 10-fold CV Pearson’s r over all cell lines, where there is data for that mark. Then for each other mark, which we name mark_2_ here, we take median Pearson’s r between the target mark and mark_2_ enrichments at TSSs of protein coding genes over all cell lines, where there is data for both, and then we take the maximum value of it. (**B**) same as (**A**), only that we consider just histone modifications, where the value for the “reference model” is still taken over all marks and not just histone modifications. (**C**) Scatter plot for median Pearson’s r comparison for each mark, where there is data for that mark available in at least two cell lines, at TSSs of protein coding genes between the 10-fold CV model performance and the correlation of the identical mark in all other cell lines. Whereas the first part is just as above, for the second one we do consider for each mark all ordered pairs of different cell lines, where we do have data for that mark in both cell lines, calculate the Pearson’s r between the enrichments at TSSs of protein coding genes in both cell lines and take the median over it. (**D**) same as (**C**), only that we consider just histone modifications.
(TIF)

S4 Fig Histogram of the mark weights in the linear model fitted for all marks on 100% of the data for each respective constellation for protein coding genes. (**A**) For TSSs in H1, (**B**) transcripts in H1, (**C**) TTSs in H1, (**D**) TSSs in H9, (**E**) transcripts in H9, (**F**) TTSs in H9, (**G**) TSSs in GM12878, (**H**) transcripts in GM12878, (**I**) TTSs in GM12878, (**J**) TSSs in IMR90, (**K**) transcripts in IMR90, (**L**) TTSs in IMR90, (**M**) TSSs in K562, (**N**) transcripts genes in K562, and (**O**) TTSs in K562.
(TIF)

S5 Fig Histogram of the mark weights in the linear models fitted for all marks on 100% of the data for each respective constellation for lincRNA genes. (**A**) For TSSs of lincRNA genes in H1, (**B**) transcripts in H1, (**C**) TTSs in H1, (**D**) TSSs in H9, (**E**) transcripts in H9, (**F**) TTSs in H9, (**G**) TSSs in GM12878, (**H**) transcripts in GM12878, (**I**) TTS in GM12878, (**J**) TSSs in IMR90, (**K**) transcripts in IMR90, (**L**) TTSs in IMR90, (**M**) TSSs in K562, (**N**) transcripts in K562, and (**O**) TTSs in K562.
(TIF)

S6 Fig Barplot of selected mark types for different mark types from the linear models fitted for all marks on 100% of the data for each respective constellation for protein coding genes. (**A**) For transcripts in H1, (**B**) TTSs in H1, (**C**) TSSs in GM12878, (**D**) transcripts in GM12878, (**E**) TTSs in GM12878, (**F**) TSSs in IMR90, (**G**) transcripts in IMR90, (**H**) TTSs in IMR90, (**I**) TSSs in K562, (**J**) transcripts in K562, and (**K**) TTSs in K562. The description of the plots is analogous to Fig 2F.
(TIF)

S7 Fig Barplot of selected mark types for different mark types from the linear models fitted for all marks on 100% of the data for each respective constellation for lincRNA genes. (**A**) For TSSs in H1, (**B**) transcripts in H1, (**C**) TTSs in H1, (**D**) TSSs in GM12878, (**E**) transcripts in GM12878, (**F**) TTSs in GM12878, (**G**) TSSs in IMR90, (**H**) transcripts in IMR90, (**I**) TTSs in IMR90, (**J**) TSSs in K562, (**K**) transcripts in K562, and (**L**) TTSs in K562. The description of the plots is analogous to Fig 2F.
(TIF)

S8 Fig Cross cell-line model performance comparison against “reference models”, where the “predictions” are the enrichments of other ChIP-seq data. (**A**) Scatter plot for median Pearson’s r comparison for each mark at TSSs of protein coding genes between the median correlation between predicted and measured values, when the models, with which the predictions are made, are fitted in other cell lines (on all marks that are present in both cell lines), and the median correlation of the identical mark in all other cell lines (the latter part is as in S3C Fig). (**B**) same as (**A**), only that we consider just histone modifications.
(TIF)

S9 Fig Cross-cell line model clustering of cell lines at different locus types. (**A**) Heatmap showing median Pearson’s r between predicted and measured values for transcripts of protein coding genes over all marks in that target cell line, that are also present in the starting cell line, where the models for the prediction are fitted in the starting cell line and then used to predict the enrichments in the target cell line. For each entry, the target cell line is named as the row entry and the starting cell line as named as the column entry. (**B**),(**C**),(**D**), and (**E**) same as (**A**) for TTS of protein coding genes, TSSs of lincRNA genes, transcripts of lincRNA genes, and TTSs of lincRNA genes, respectively.
(TIF)

S10 Fig Model performance for CAGE for lincRNA genes. Barplot of Pearson’s r (when using 10-fold CV) for different models for lincRNA genes. The models are indexed analogously to Fig 4B and for each of these the pseudocount *ε* was optimized.
(TIF)

S11 Fig Cross-cell line performance for CAGE models. Barplot of Pearson’s r, when considering each possible ordered pair of different cell lines, shown in blue, and the Pearson’s r (when using 10-fold CV) for individual cell lines, shown in red, when using linear models on 40-bin resolution for protein coding genes (**A**), MARS models on 40-bin resolution (middle two bins) for protein coding genes (**B**), linear models on 40-bin resolution (middle two bins) for protein coding genes (**C**), MARS models on 1-bin resolution for protein coding genes (**D**), linear models on 1-bin resolution for protein coding genes (**E**), MARS models on 40-bin resolution for lincRNA genes (**F**), linear models on 40-bin resolution for lincRNA genes (**G**), MARS models on 40-bin resolution (middle two bins) for lincRNA genes (**H**), linear models on 40-bin resolution (middle two bins) for lincRNA genes (**I**), MARS models on 1-bin resolution for lincRNA genes (**J**), and linear models on 1-bin resolution for lincRNA genes (**K**). The description of the plots is analogous to Fig 4D.
(TIF)

S12 Fig 10-fold CV IHEC model performance. (**A**) Histogram of Pearson’s r over all other marks between predicted and measured values (when using 10-fold CV) over all cell lines (where data was available for this mark) around the TSSs of protein coding genes. (**B**) Histogram of Pearson’s r over all cell lines, all other histone modifications (where data for these marks was available for this cell line), and all locus constellations. (**C**) Histogram of Pearson’s r over all cell lines, marks that are not histone modifications (where data for these marks was available for this cell line), and all locus constellations.
(TIF)

S13 Fig 10-fold CV IHEC model performance comparison against “reference models”, where the “predictions” are the enrichments of other ChIP-seq data or 10-fold CV models fitted on all marks. (**A**) Scatter plot for median Pearson’s r comparison for each histone modification (apart from the six IHEC histone modifications) at TSSs of protein coding genes between the 10-fold CV model performance, where the models are fitted on all other marks, and the 10-fold CV model performance, where the models are fitted on IHEC marks. (**B**) Scatter plot for median Pearson’s r comparison for each mark (apart from the six IHEC histone modifications), where there is data for that mark available in at least two cell lines, at TSSs of protein coding genes between the 10-fold CV model performance, where the models are fitted on IHEC marks, and the median correlation of the identical mark in all other cell lines (as in S3 Fig). (**C**) same as (**B**), only that we consider just histone modifications (apart from the six IHEC histone modifications).
(TIF)

S14 Fig Cross cell-line model IHEC performance comparison. (**A**) Scatter plot for median Pearson’s r comparison for each histone modification (apart from the six IHEC histone modifications), where there is data for at least two cell lines available, at TSSs of protein coding genes between the median 10-fold CV model performance, where the models are fitted on IHEC marks, and the median correlation between predicted and measured values, when the models, with which the predictions are made, are fitted in other cell lines on the IHEC marks. (**B**) Scatter plot for median Pearson’s r comparison for each mark (apart from the six IHEC histone modifications), where there is data for at least two cell lines available, at TSSs of protein coding genes between the correlation between predicted and measured values, when the models, with which the predictions are made, are fitted in other cell lines on IHEC marks, and the median correlation of the identical mark in all other cell lines (as in S8 Fig). (**C**) same as (**B**), only that we consider just histone modifications. (**D**) Scatter plot for median Pearson’s r comparison for each mark (apart from the six IHEC histone modifications), where there is data for at least two cell lines available, at TSSs of protein coding genes between the median correlation between predicted and measured values, when the models, with which the predictions are made, are fitted in other cell lines on the IHEC marks, and the median correlation between predicted and measured values, when the models, with which the predictions are made, are fitted in other cell lines on all marks, that are present in both cell lines. (**E**) same as (**D**), only that we consider just histone modifications.
(TIF)

S15 Fig Cross-cell line IHEC model clustering of cell lines at different locus types. (**A**) Heatmap showing median Pearson’s r between predicted and measured values for transcripts of protein coding genes over all marks for which there is data available in all cell lines apart from the IHEC marks (i.e., DNase hypersensitivity, H2.AZ, H3K4me2, H3K9ac, H3K79me2, H4K20me1), where the models for the prediction are fitted on the IHEC marks in the starting cell line and then used to predict the enrichments in the target cell line. For each entry, the target cell line is named as the row entry and the starting cell line as named as the column entry. (**B**),(**C**),(**D**), and (**E**) same as (**A**) for TTS of protein coding genes, TSSs of lincRNA genes, transcripts of lincRNA genes, and TTSs of lincRNA genes, respectively.
(TIF)

S16 Fig Barplot of median Pearson’s r for a particular mark over the measured and predicted values for all other marks in the region around TSSs of protein coding genes. (**A**) For GM12878, (**B**) H1, (**C**) IMR90, and (**D**) K562. The description of the plots is analogous to Fig 6A.
(TIF)

S17 Fig Number of marks that are needed in order to exceed a given median Pearson’s r threshold. (**A**) For GM12878, (**B**) H1, (**C**) H9, and (**D**) IMR90. The description of the plots is analogous to Fig 6C.
(TIF)

## Abbreviations

bp: base pairs
CAGE: cap analysis gene expression
ChIP-seq: chromatin immunoprecipitation followed by high-throughput DNA sequencing
CV: cross-validation
GEx: gene expression
kb: kilobases
MARS: multivariate adaptive regression spline
Pearson’s r: Pearson correlation coefficient
SI: supporting information
TSS: transcription start site
TTS: transcription termination site
