# Supplementary figures and images for: Transcription factors, coregulators, and epigenetic marks are linearly correlated and highly redundant
Source: PLoS One. 2017 Dec 7;12(12):e0186324. doi: 10.1371/journal.pone.0186324 (PMC5720766; doi:10.1371/journal.pone.0186324)

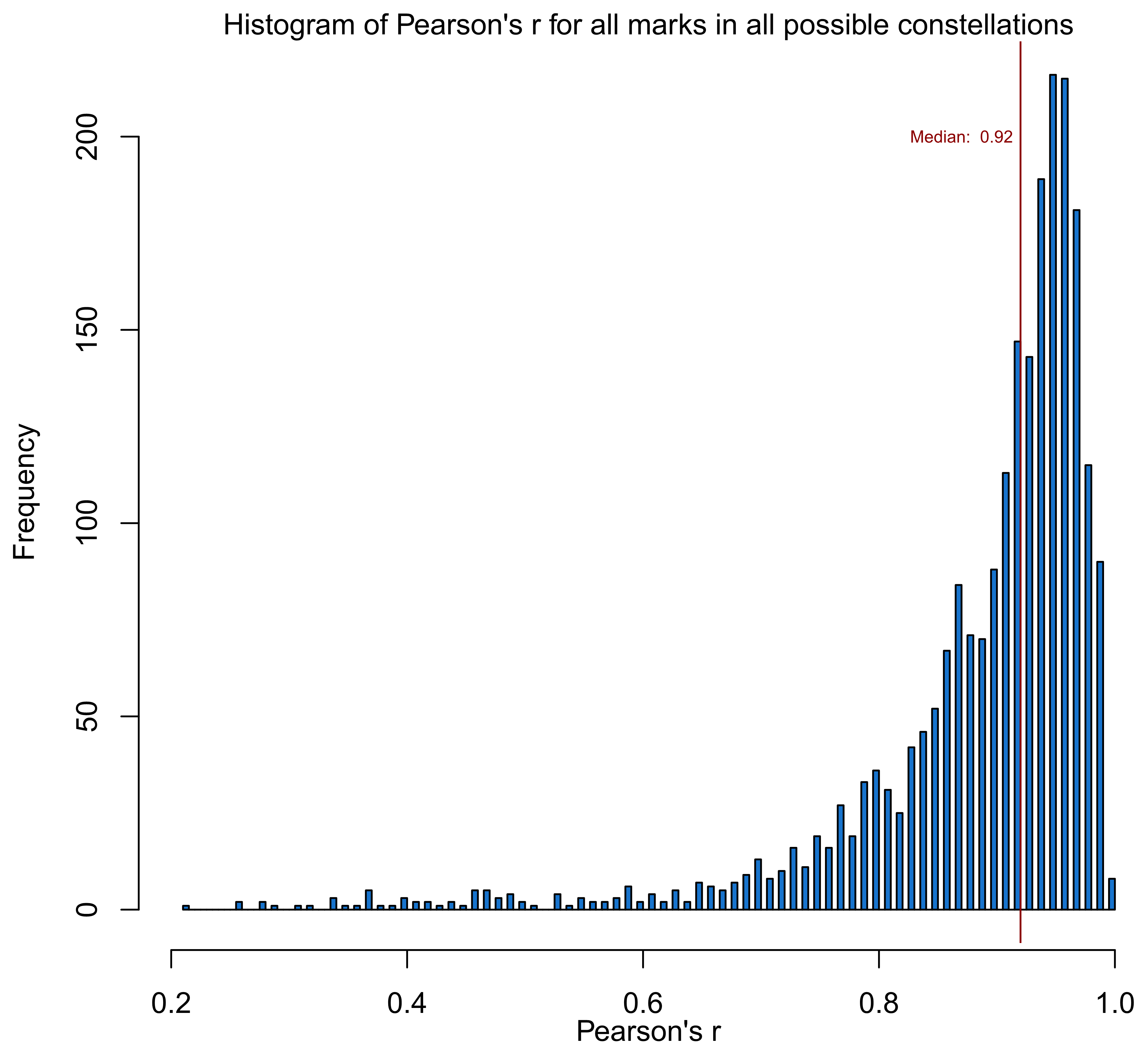

Supplement: S1 Fig — Histogram of Pearson’s r between measured and predicted values (when using 10-fold CV) for all marks and constellations. (TIF) [file pone.0186324.s002.tif]

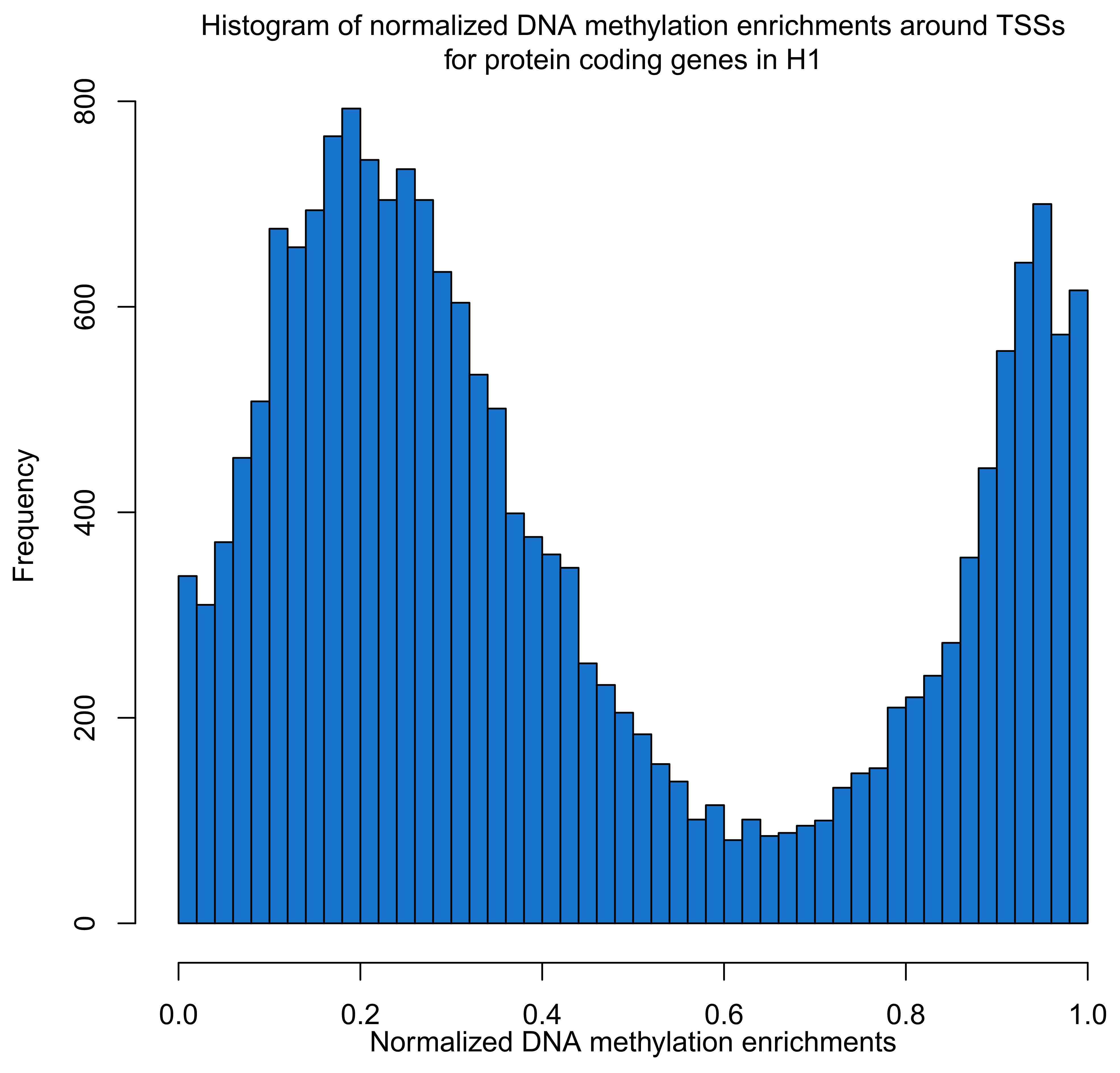

Supplement: S2 Fig — Histogram of normalized DNA methylation enrichments in the regions around TSSs of protein coding genes in H1. Here a value of 0 means that 0% of the CpGs are methylated, and a value of 1 means that 100% of the CpGs are methylated. (TIF) [file pone.0186324.s003.tif]

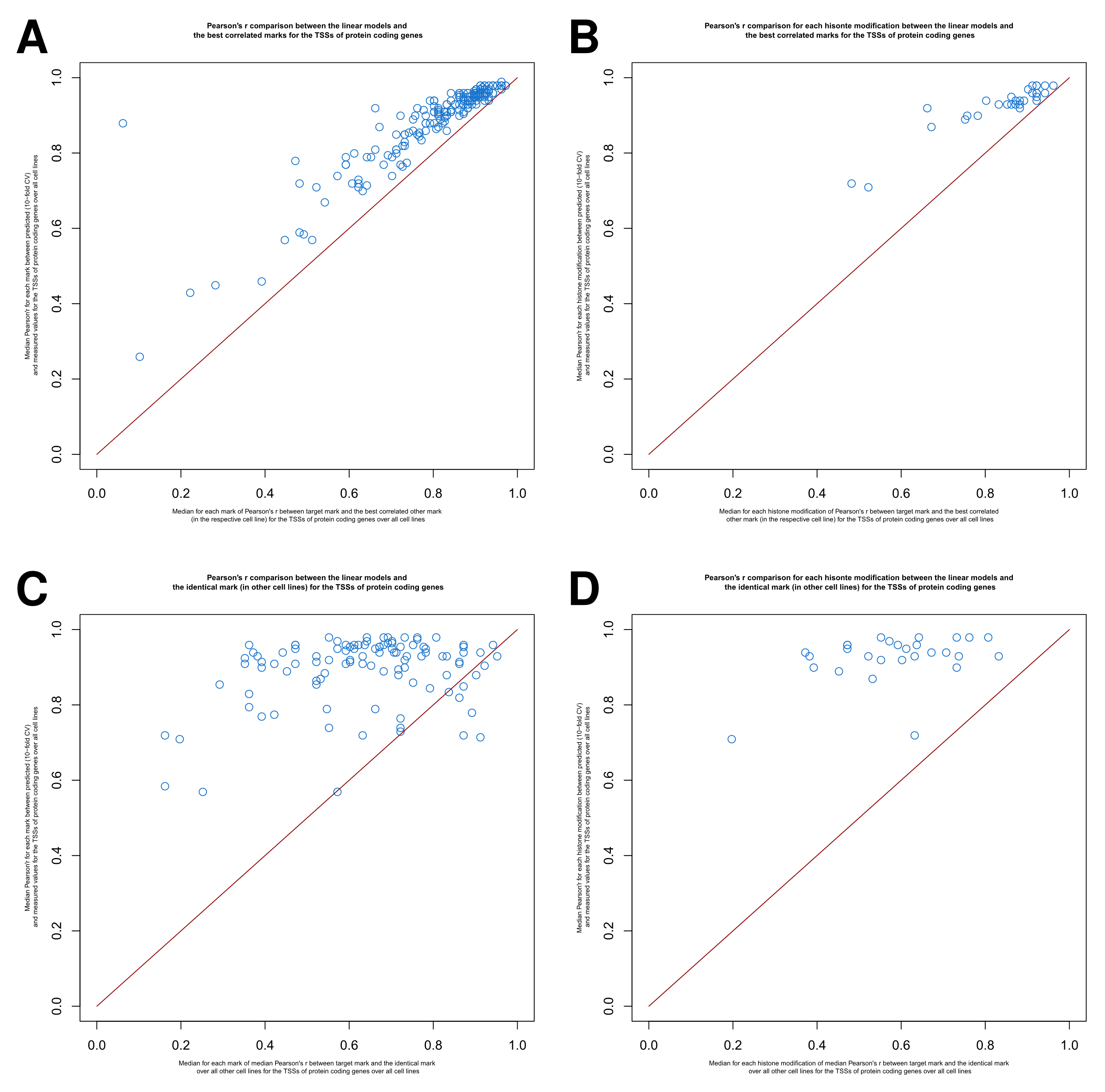

Supplement: S3 Fig — (A) Scatter plot for median Pearson’s r comparison for each mark at TSSs of protein coding genes between the 10-fold CV model performance and the correlation of the best correlated mark in the same respective cell line. That means for each mark we take the median 10-fold CV Pearson’s r over all cell lines, where there is data for that mark. Then for each other mark, which we name mark2 here, we take median Pearson’s r between the target mark and mark2 enrichments at TSSs of protein coding genes over all cell lines, where there is data for both, and then we take the maximum value of it. (B) same as (A), only that we consider just histone modifications, where the value for the “reference model” is still taken over all marks and not just histone modifications. (C) Scatter plot for median Pearson’s r comparison for each mark, where there is data for that mark available in at least two cell lines, at TSSs of protein coding genes between the 10-fold CV model performance and the correlation of the identical mark in all other cell lines. Whereas the first part is just as above, for the second one we do consider for each mark all ordered pairs of different cell lines, where we do have data for that mark in both cell lines, calculate the Pearson’s r between the enrichments at TSSs of protein coding genes in both cell lines and take the median over it. (D) same as (C), only that we consider just histone modifications. (TIF) [file pone.0186324.s004.tif]

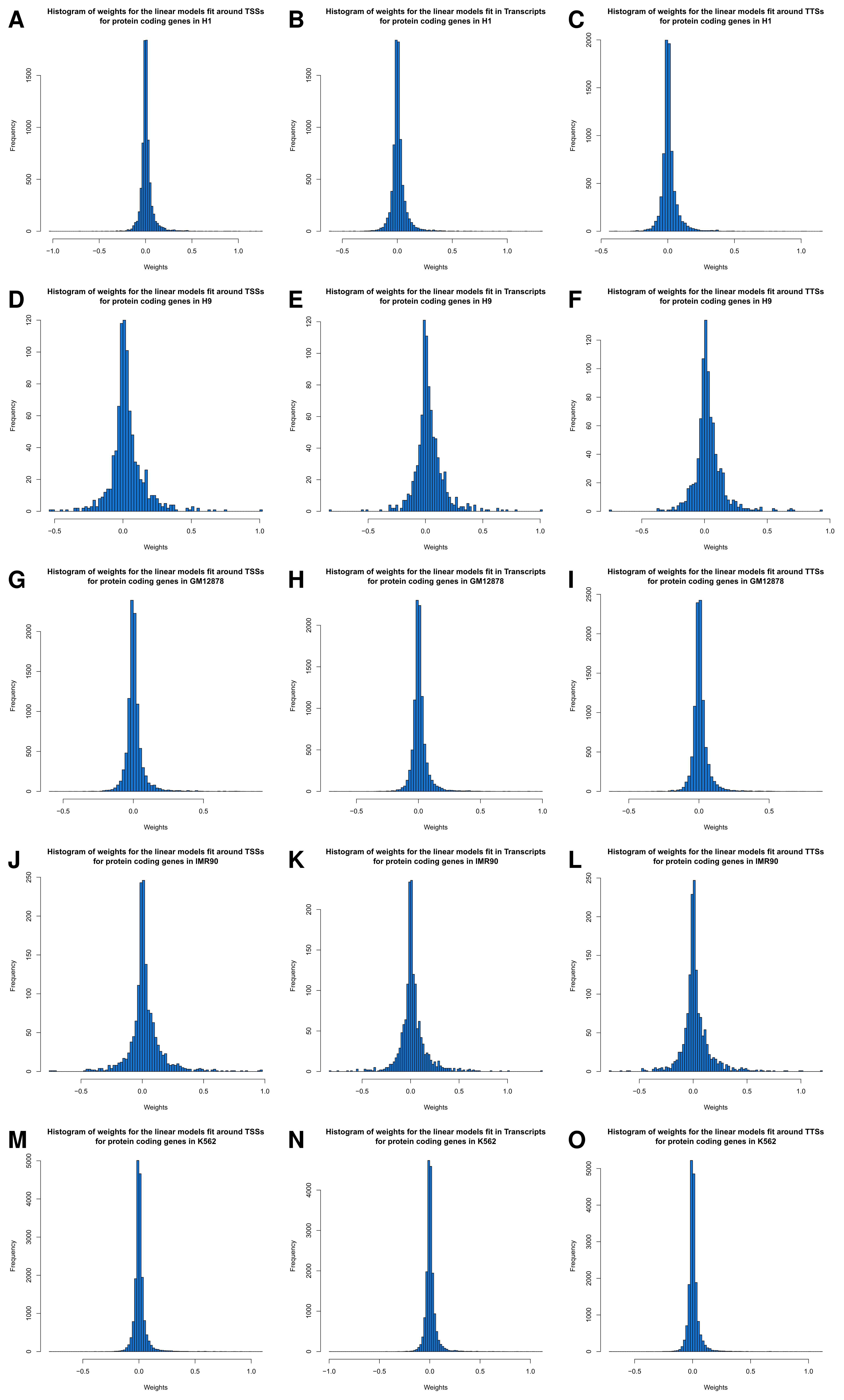

Supplement: S4 Fig — (A) For TSSs in H1, (B) transcripts in H1, (C) TTSs in H1, (D) TSSs in H9, (E) transcripts in H9, (F) TTSs in H9, (G) TSSs in GM12878, (H) transcripts in GM12878, (I) TTSs in GM12878, (J) TSSs in IMR90, (K) transcripts in IMR90, (L) TTSs in IMR90, (M) TSSs in K562, (N) transcripts genes in K562, and (O) TTSs in K562. (TIF) [file pone.0186324.s005.tif]

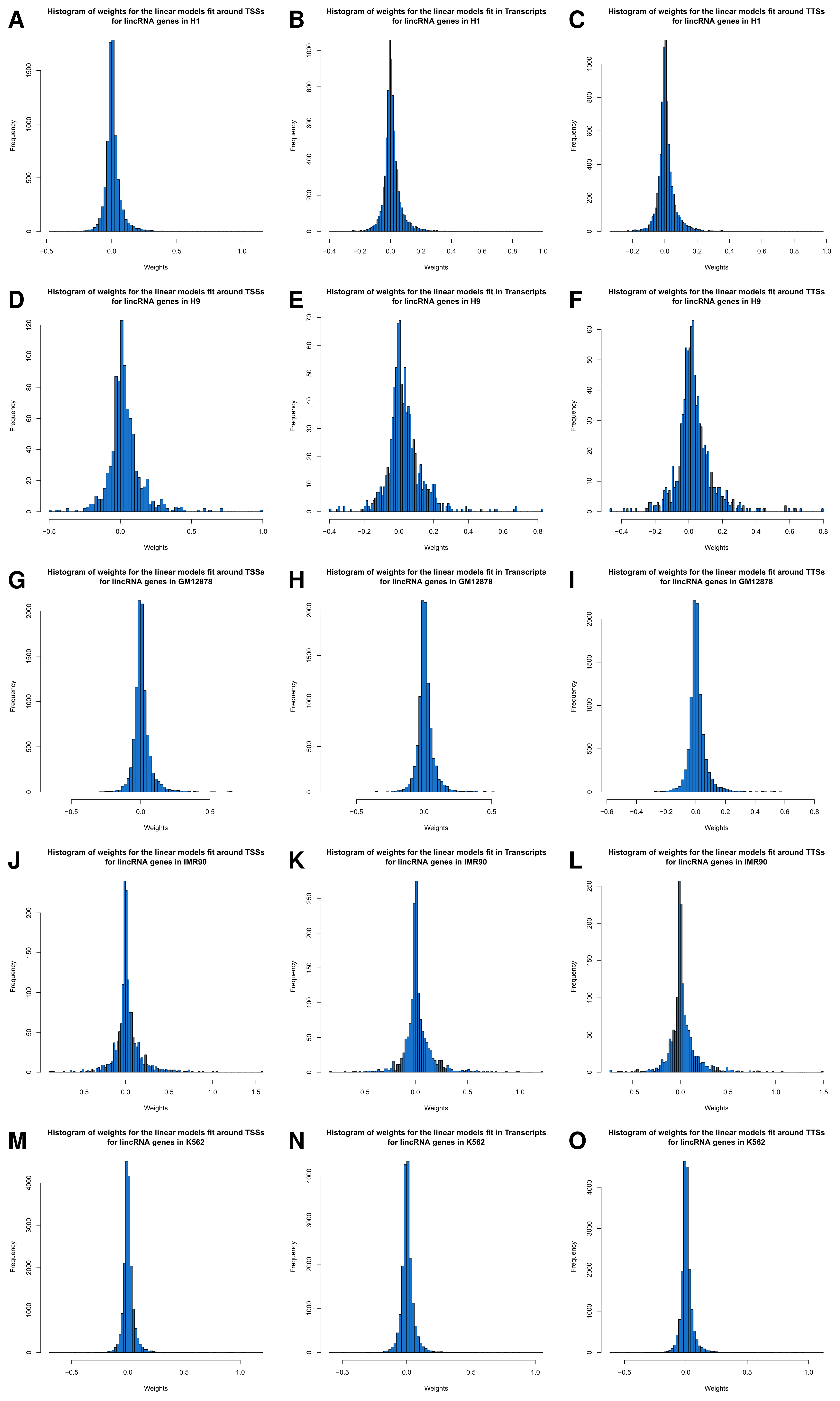

Supplement: S5 Fig — (A) For TSSs of lincRNA genes in H1, (B) transcripts in H1, (C) TTSs in H1, (D) TSSs in H9, (E) transcripts in H9, (F) TTSs in H9, (G) TSSs in GM12878, (H) transcripts in GM12878, (I) TTS in GM12878, (J) TSSs in IMR90, (K) transcripts in IMR90, (L) TTSs in IMR90, (M) TSSs in K562, (N) transcripts in K562, and (O) TTSs in K562. (TIF) [file pone.0186324.s006.tif]

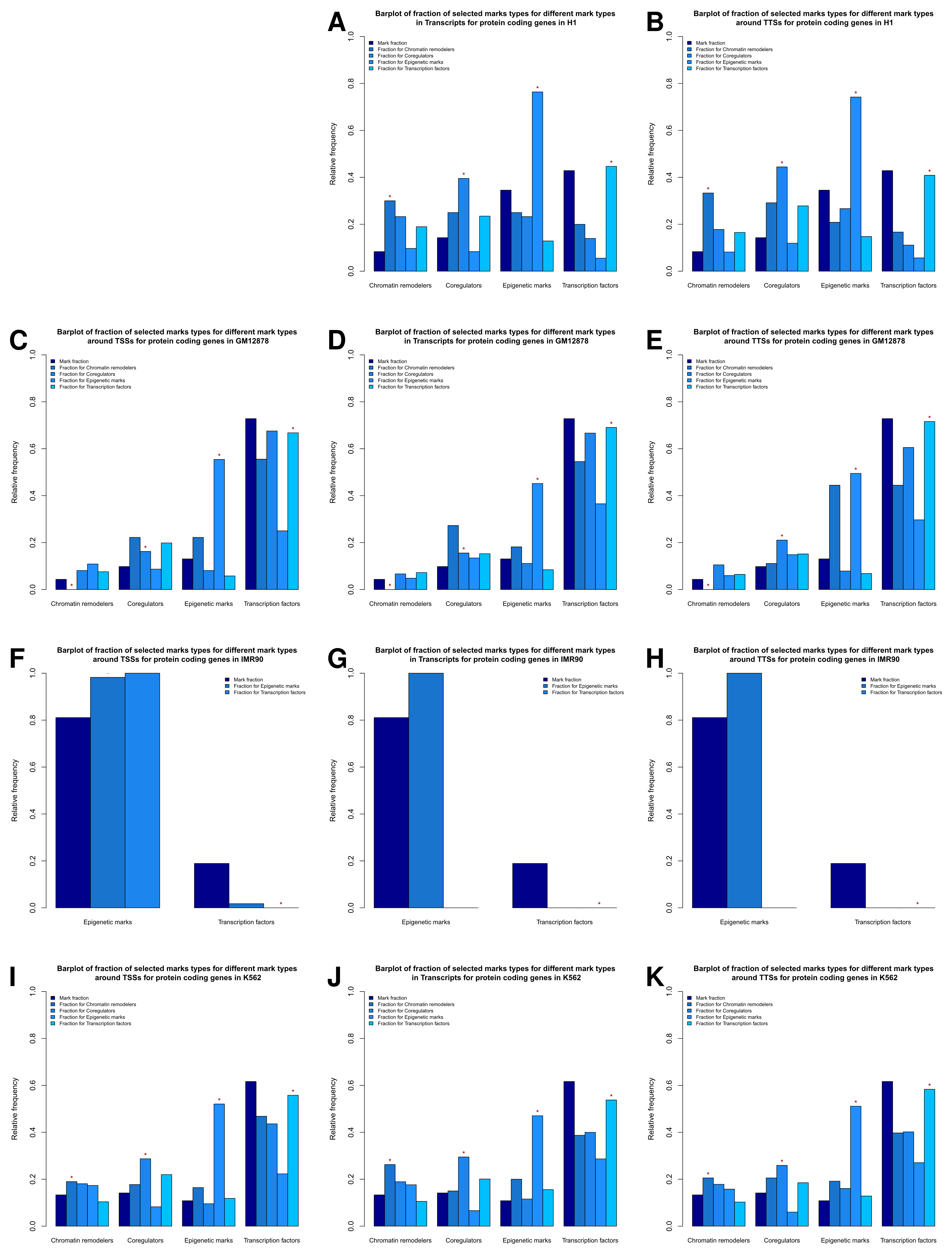

Supplement: S6 Fig — (A) For transcripts in H1, (B) TTSs in H1, (C) TSSs in GM12878, (D) transcripts in GM12878, (E) TTSs in GM12878, (F) TSSs in IMR90, (G) transcripts in IMR90, (H) TTSs in IMR90, (I) TSSs in K562, (J) transcripts in K562, and (K) TTSs in K562. The description of the plots is analogous to Fig 2F. (TIF) [file pone.0186324.s007.tif]

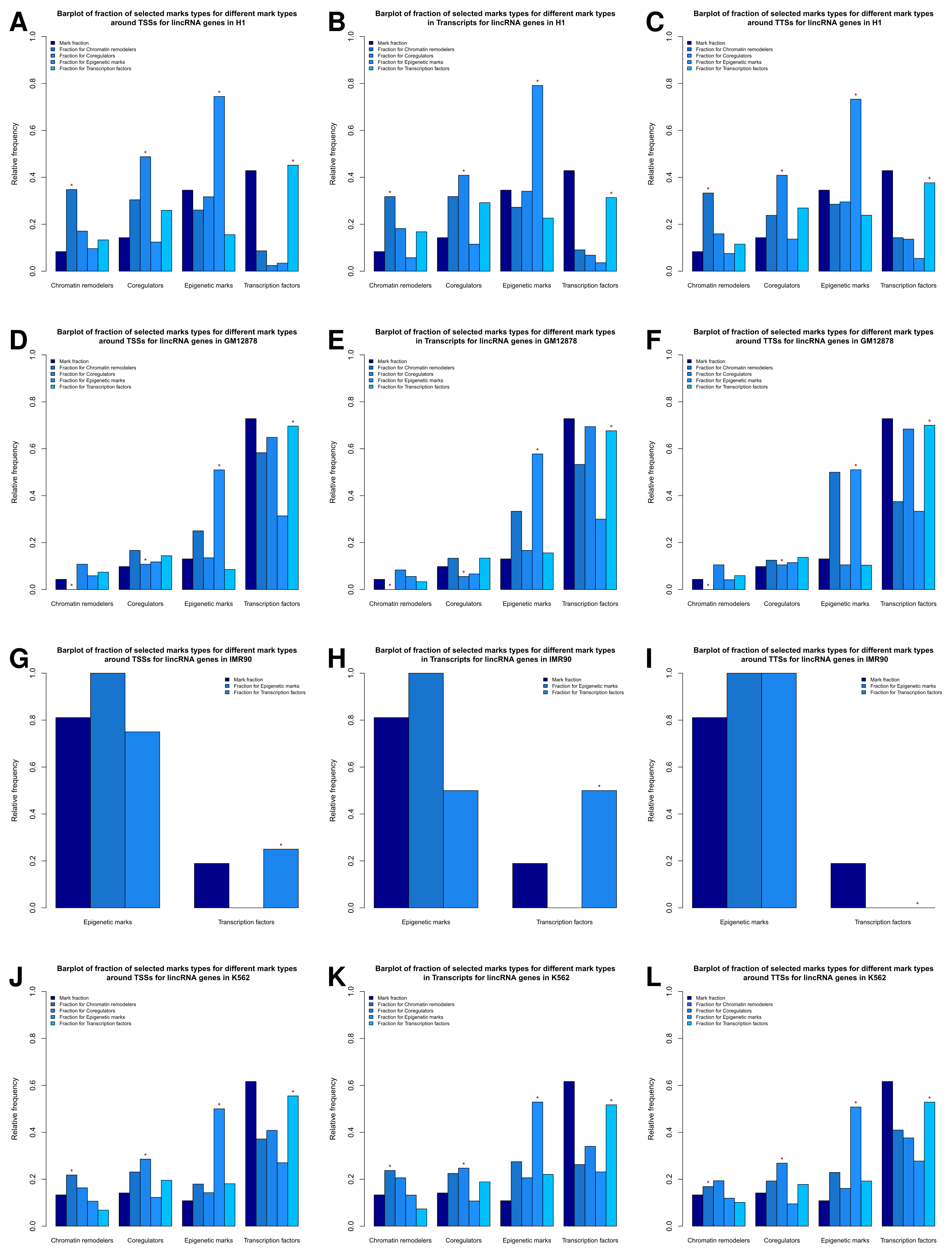

Supplement: S7 Fig — (A) For TSSs in H1, (B) transcripts in H1, (C) TTSs in H1, (D) TSSs in GM12878, (E) transcripts in GM12878, (F) TTSs in GM12878, (G) TSSs in IMR90, (H) transcripts in IMR90, (I) TTSs in IMR90, (J) TSSs in K562, (K) transcripts in K562, and (L) TTSs in K562. The description of the plots is analogous to Fig 2F. (TIF) [file pone.0186324.s008.tif]

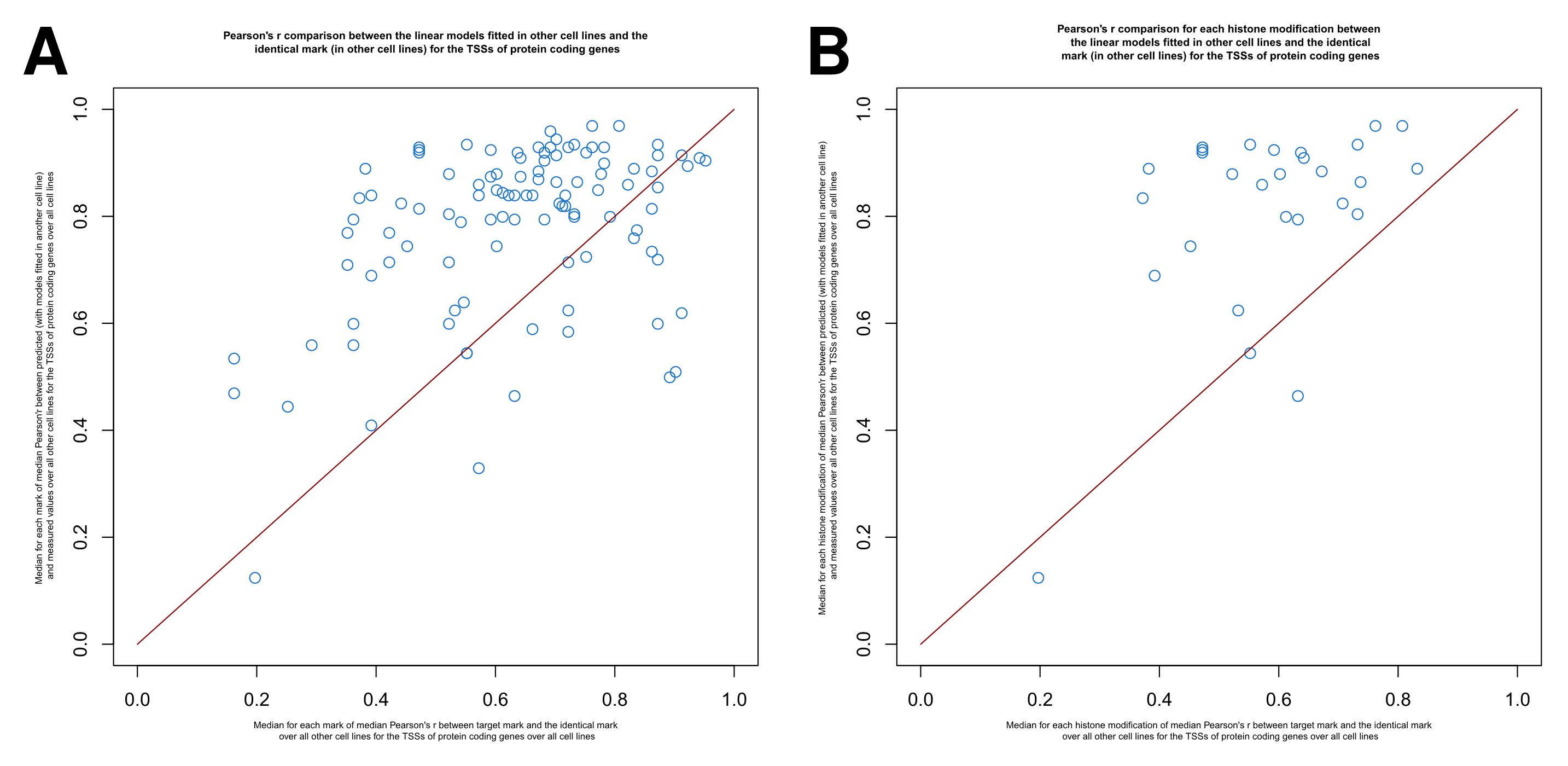

Supplement: S8 Fig — (A) Scatter plot for median Pearson’s r comparison for each mark at TSSs of protein coding genes between the median correlation between predicted and measured values, when the models, with which the predictions are made, are fitted in other cell lines (on all marks that are present in both cell lines), and the median correlation of the identical mark in all other cell lines (the latter part is as in S3C Fig). (B) same as (A), only that we consider just histone modifications. (TIF) [file pone.0186324.s009.tif]

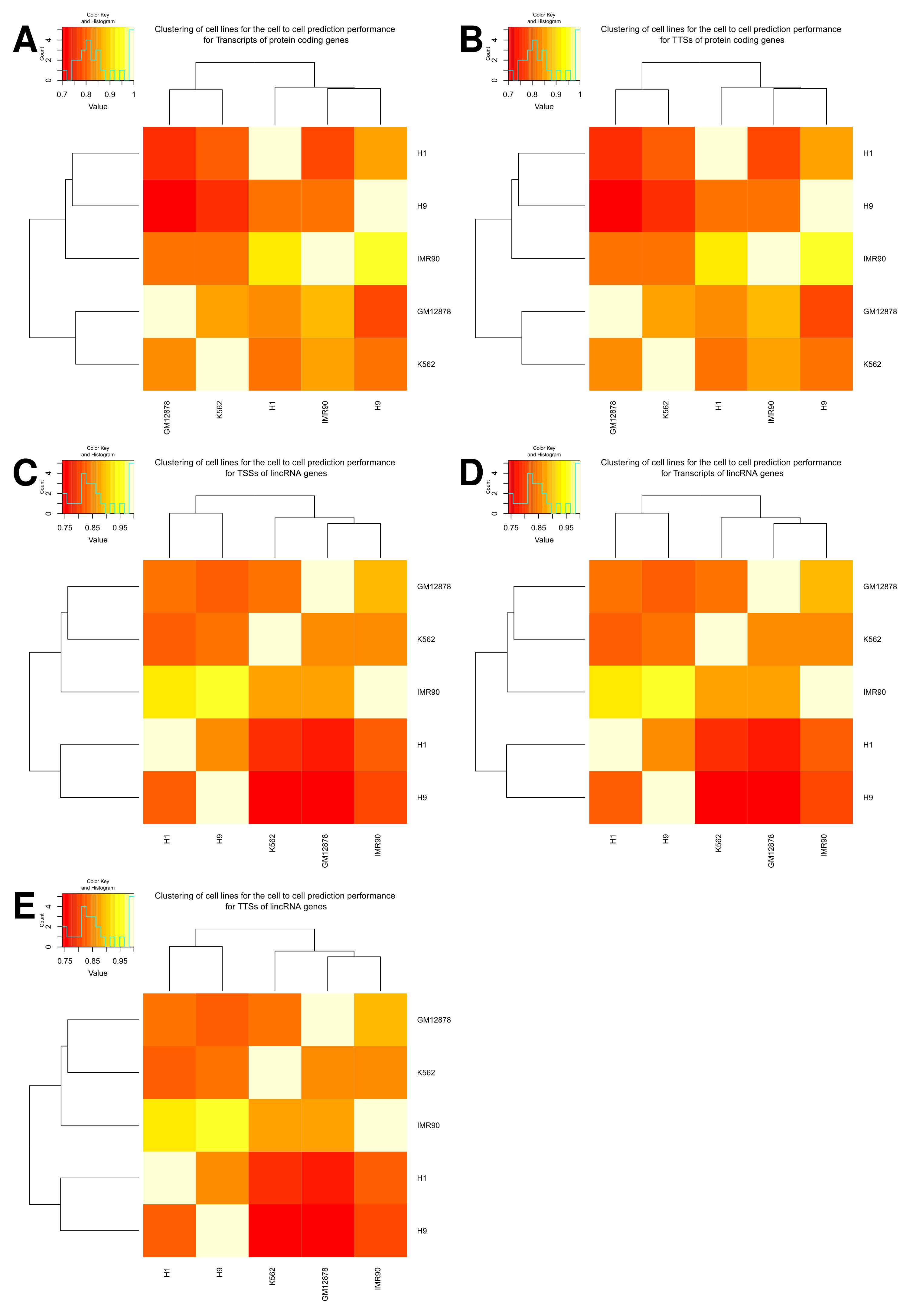

Supplement: S9 Fig — (A) Heatmap showing median Pearson’s r between predicted and measured values for transcripts of protein coding genes over all marks in that target cell line, that are also present in the starting cell line, where the models for the prediction are fitted in the starting cell line and then used to predict the enrichments in the target cell line. For each entry, the target cell line is named as the row entry and the starting cell line as named as the column entry. (B),(C),(D), and (E) same as (A) for TTS of protein coding genes, TSSs of lincRNA genes, transcripts of lincRNA genes, and TTSs of lincRNA genes, respectively. (TIF) [file pone.0186324.s010.tif]

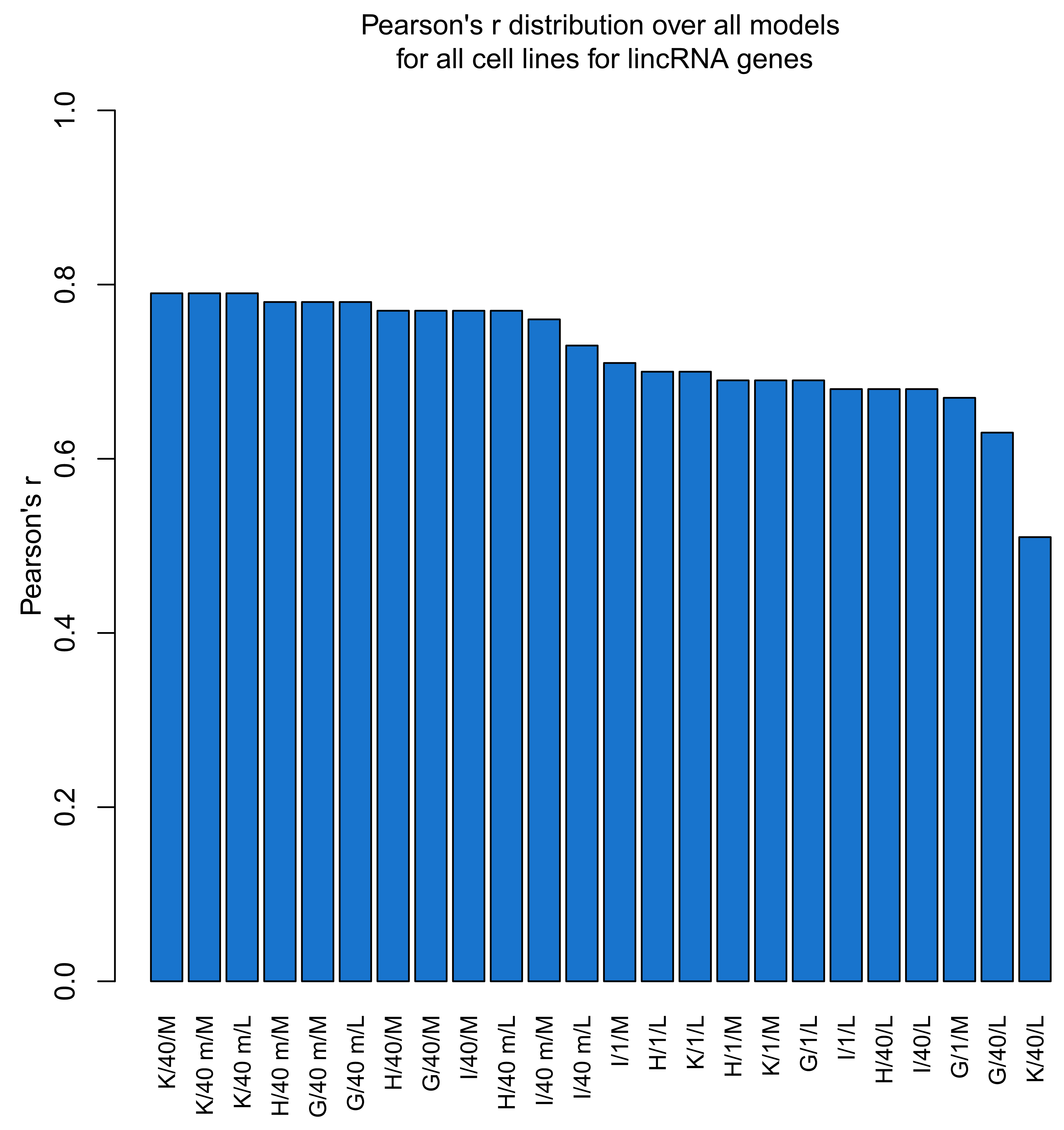

Supplement: S10 Fig — Barplot of Pearson’s r (when using 10-fold CV) for different models for lincRNA genes. The models are indexed analogously to Fig 4B and for each of these the pseudocount ε was optimized. (TIF) [file pone.0186324.s011.tif]

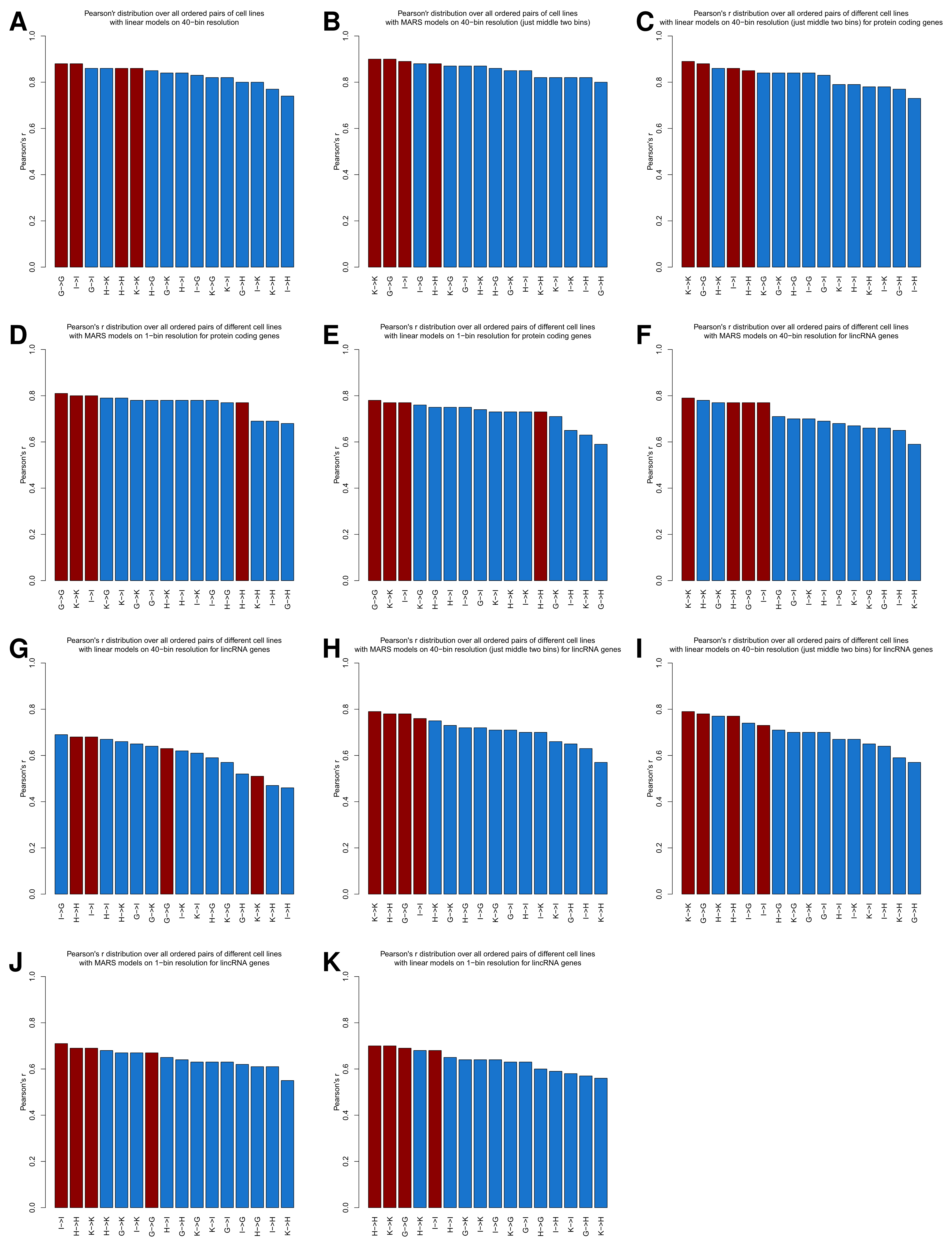

Supplement: S11 Fig — Barplot of Pearson’s r, when considering each possible ordered pair of different cell lines, shown in blue, and the Pearson’s r (when using 10-fold CV) for individual cell lines, shown in red, when using linear models on 40-bin resolution for protein coding genes (A), MARS models on 40-bin resolution (middle two bins) for protein coding genes (B), linear models on 40-bin resolution (middle two bins) for protein coding genes (C), MARS models on 1-bin resolution for protein coding genes (D), linear models on 1-bin resolution for protein coding genes (E), MARS models on 40-bin resolution for lincRNA genes (F), linear models on 40-bin resolution for lincRNA genes (G), MARS models on 40-bin resolution (middle two bins) for lincRNA genes (H), linear models on 40-bin resolution (middle two bins) for lincRNA genes (I), MARS models on 1-bin resolution for lincRNA genes (J), and linear models on 1-bin resolution for lincRNA genes (K). The description of the plots is analogous to Fig 4D. (TIF) [file pone.0186324.s012.tif]

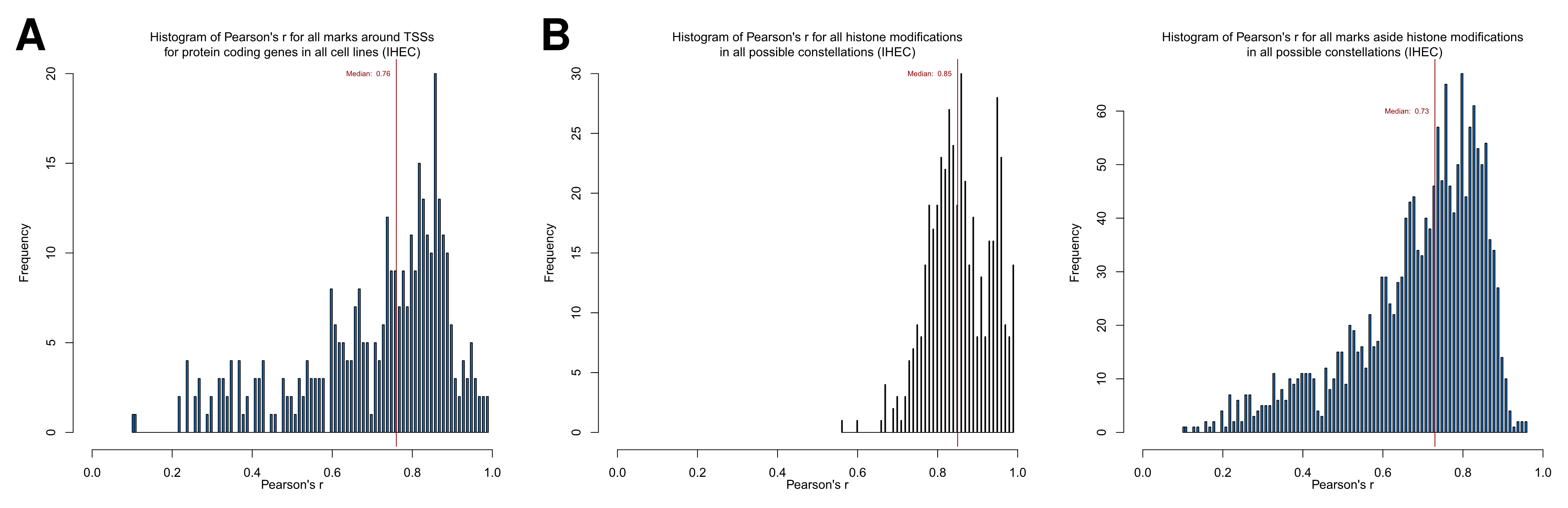

Supplement: S12 Fig — (A) Histogram of Pearson’s r over all other marks between predicted and measured values (when using 10-fold CV) over all cell lines (where data was available for this mark) around the TSSs of protein coding genes. (B) Histogram of Pearson’s r over all cell lines, all other histone modifications (where data for these marks was available for this cell line), and all locus constellations. (C) Histogram of Pearson’s r over all cell lines, marks that are not histone modifications (where data for these marks was available for this cell line), and all locus constellations. (TIF) [file pone.0186324.s013.tif]

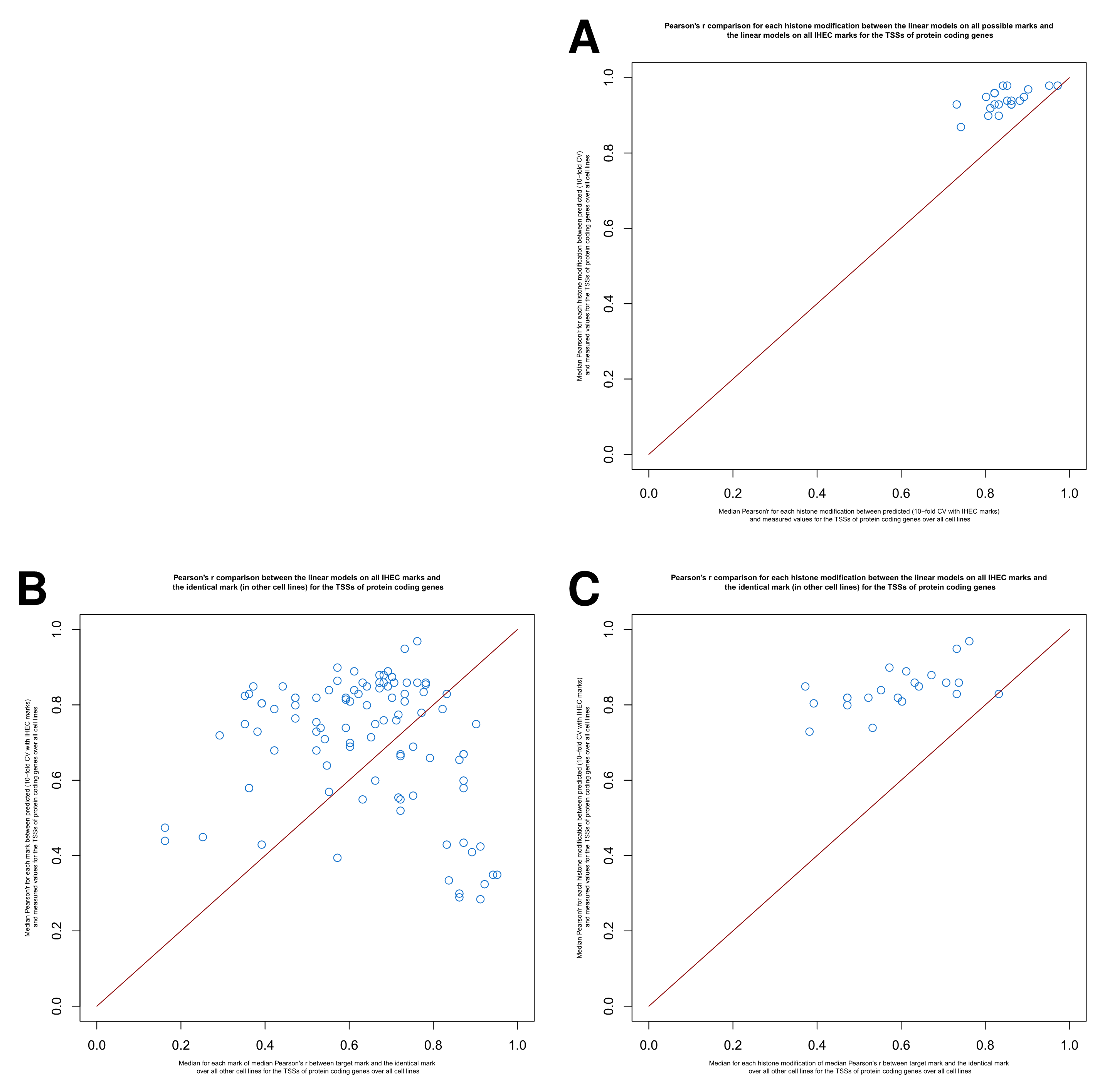

Supplement: S13 Fig — (A) Scatter plot for median Pearson’s r comparison for each histone modification (apart from the six IHEC histone modifications) at TSSs of protein coding genes between the 10-fold CV model performance, where the models are fitted on all other marks, and the 10-fold CV model performance, where the models are fitted on IHEC marks. (B) Scatter plot for median Pearson’s r comparison for each mark (apart from the six IHEC histone modifications), where there is data for that mark available in at least two cell lines, at TSSs of protein coding genes between the 10-fold CV model performance, where the models are fitted on IHEC marks, and the median correlation of the identical mark in all other cell lines (as in S3 Fig). (C) same as (B), only that we consider just histone modifications (apart from the six IHEC histone modifications). (TIF) [file pone.0186324.s014.tif]

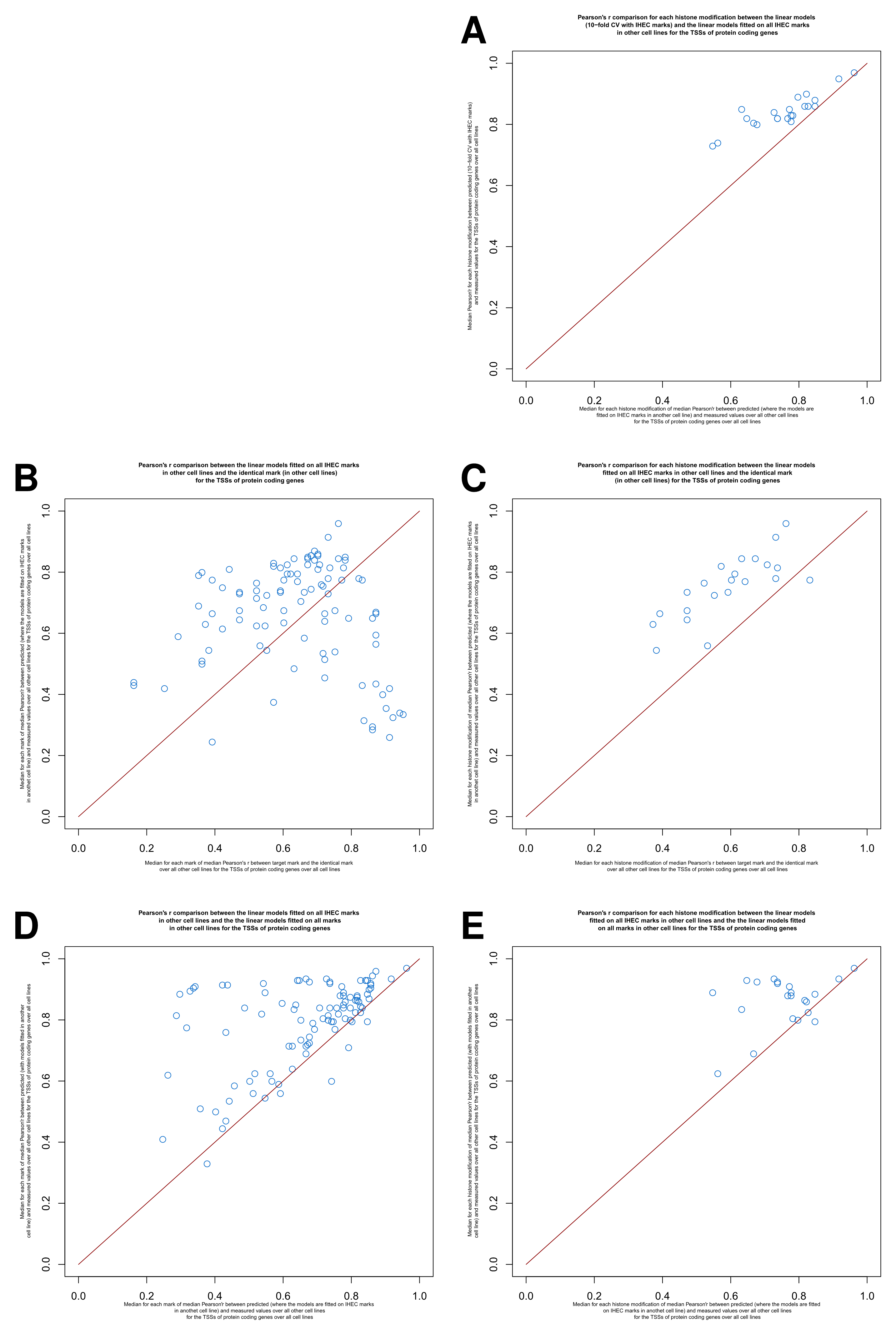

Supplement: S14 Fig — (A) Scatter plot for median Pearson’s r comparison for each histone modification (apart from the six IHEC histone modifications), where there is data for at least two cell lines available, at TSSs of protein coding genes between the median 10-fold CV model performance, where the models are fitted on IHEC marks, and the median correlation between predicted and measured values, when the models, with which the predictions are made, are fitted in other cell lines on the IHEC marks. (B) Scatter plot for median Pearson’s r comparison for each mark (apart from the six IHEC histone modifications), where there is data for at least two cell lines available, at TSSs of protein coding genes between the correlation between predicted and measured values, when the models, with which the predictions are made, are fitted in other cell lines on IHEC marks, and the median correlation of the identical mark in all other cell lines (as in S8 Fig). (C) same as (B), only that we consider just histone modifications. (D) Scatter plot for median Pearson’s r comparison for each mark (apart from the six IHEC histone modifications), where there is data for at least two cell lines available, at TSSs of protein coding genes between the median correlation between predicted and measured values, when the models, with which the predictions are made, are fitted in other cell lines on the IHEC marks, and the median correlation between predicted and measured values, when the models, with which the predictions are made, are fitted in other cell lines on all marks, that are present in both cell lines. (E) same as (D), only that we consider just histone modifications. (TIF) [file pone.0186324.s015.tif]

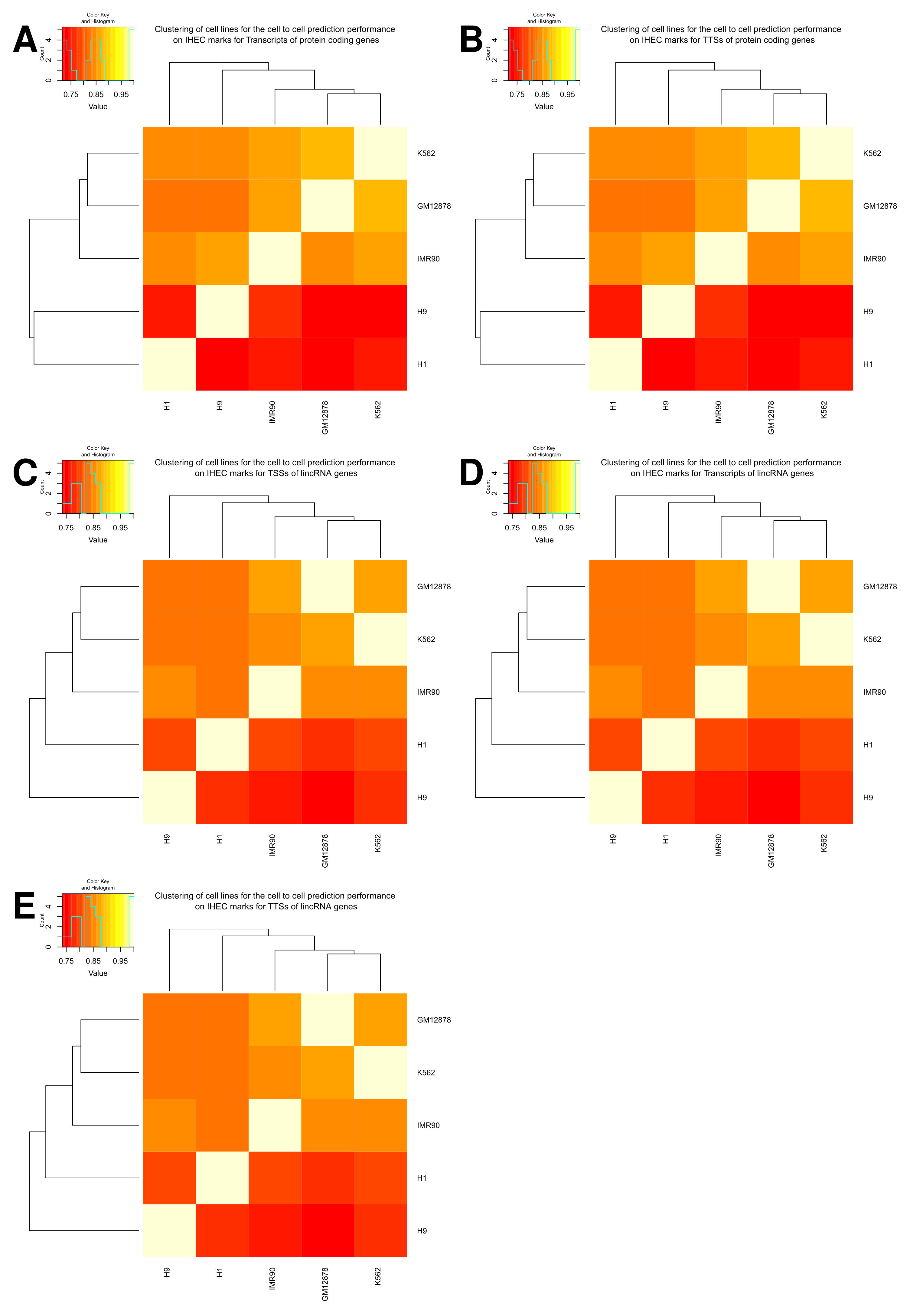

Supplement: S15 Fig — (A) Heatmap showing median Pearson’s r between predicted and measured values for transcripts of protein coding genes over all marks for which there is data available in all cell lines apart from the IHEC marks (i.e., DNase hypersensitivity, H2.AZ, H3K4me2, H3K9ac, H3K79me2, H4K20me1), where the models for the prediction are fitted on the IHEC marks in the starting cell line and then used to predict the enrichments in the target cell line. For each entry, the target cell line is named as the row entry and the starting cell line as named as the column entry. (B),(C),(D), and (E) same as (A) for TTS of protein coding genes, TSSs of lincRNA genes, transcripts of lincRNA genes, and TTSs of lincRNA genes, respectively. (TIF) [file pone.0186324.s016.tif]

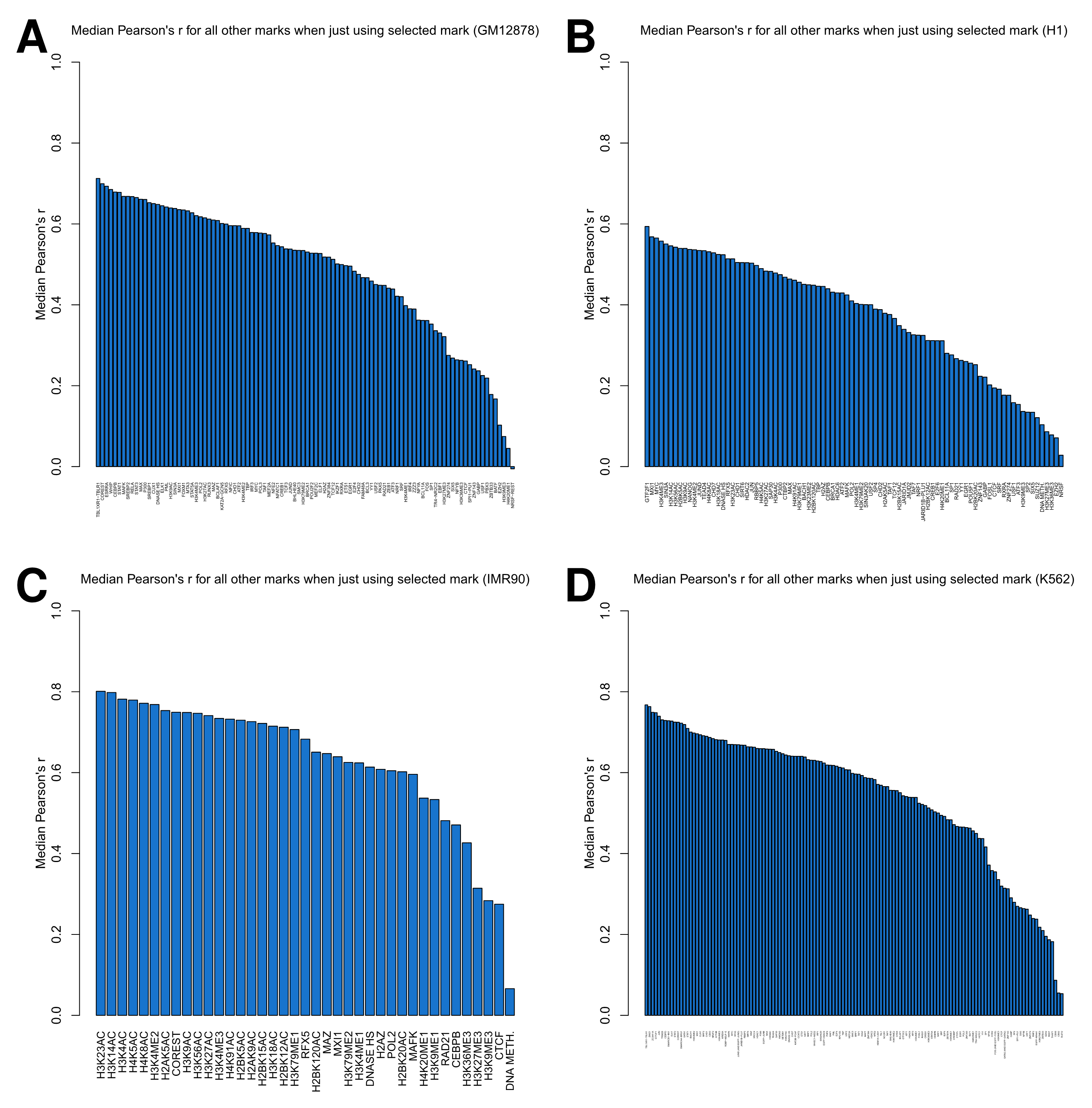

Supplement: S16 Fig — (A) For GM12878, (B) H1, (C) IMR90, and (D) K562. The description of the plots is analogous to Fig 6A. (TIF) [file pone.0186324.s017.tif]

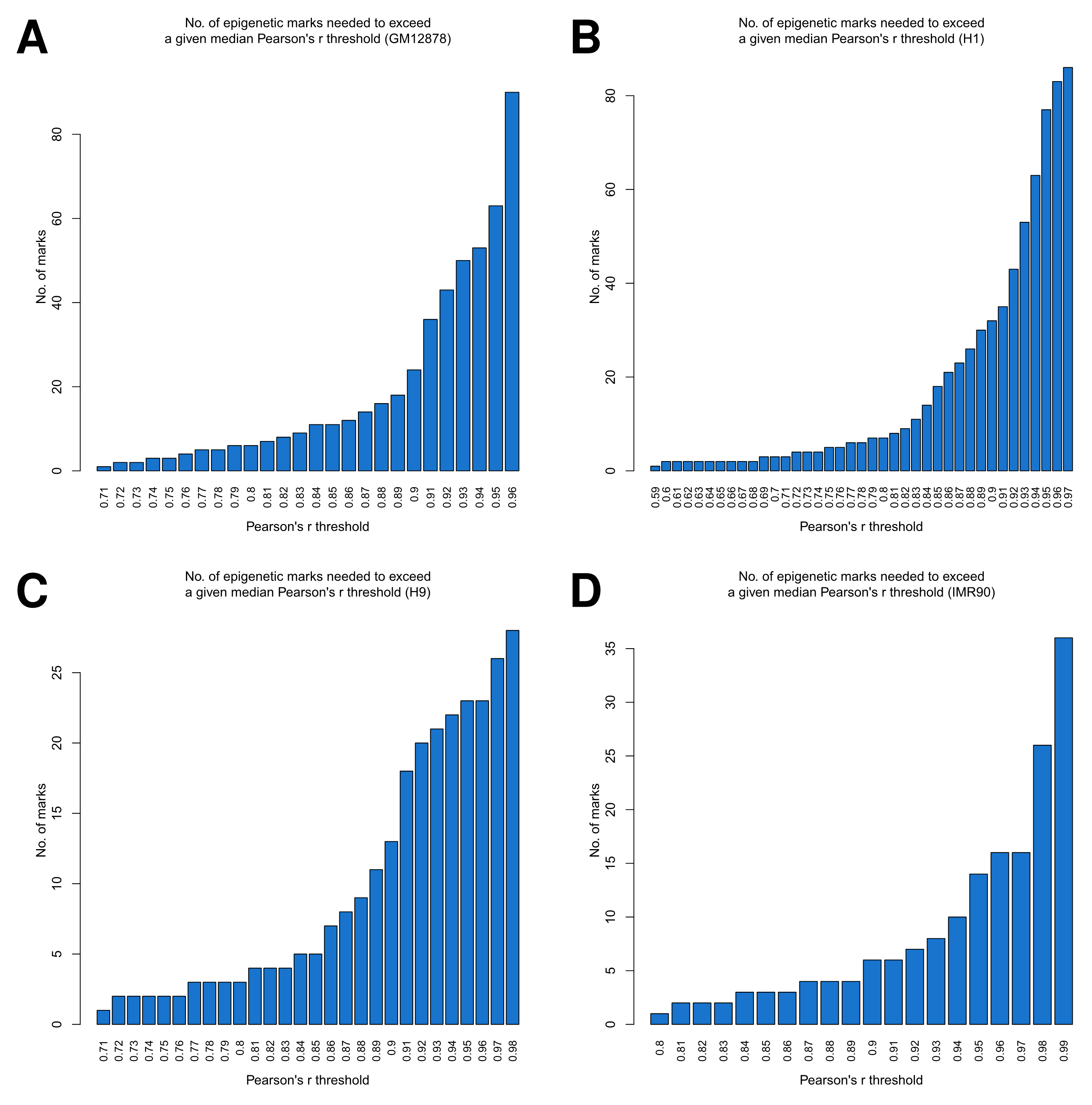

Supplement: S17 Fig — (A) For GM12878, (B) H1, (C) H9, and (D) IMR90. The description of the plots is analogous to Fig 6C. (TIF) [file pone.0186324.s018.tif]
